# CRISPR/Cas9‐based genome editing of 14 lipid metabolic genes reveals a sporopollenin metabolon ZmPKSB‐ZmTKPR1‐1/‐2 required for pollen exine formation in maize

**DOI:** 10.1111/pbi.14181

**Published:** 2023-10-04

**Authors:** Xueli An, Shaowei Zhang, Yilin Jiang, Xinze Liu, Chaowei Fang, Jing Wang, Lina Zhao, Quancan Hou, Juan Zhang, Xiangyuan Wan

**Affiliations:** ^1^ Research Institute of Biology and Agriculture University of Science and Technology Beijing Beijing China; ^2^ Industry Research Institute of Biotechnology Breeding Yili Normal University Yining China; ^3^ Zhongzhi International Institute of Agricultural Biosciences Beijing China; ^4^ Beijing Engineering Laboratory of Main Crop Bio‐Tech Breeding, Beijing International Science and Technology Cooperation Base of Bio‐Tech Breeding Beijing Solidwill Sci‐Tech Co. Ltd. Beijing China

**Keywords:** CRISPR/Cas9, *ZmTKPR1‐1* and *ZmTKPR1‐2*, sporopollenin metabolon, anther and pollen development, male sterility, maize (*Zea mays*)

## Abstract

Lipid biosynthesis and transport are essential for plant male reproduction. Compared with *Arabidopsis* and rice, relatively fewer maize lipid metabolic genic male‐sterility (GMS) genes have been identified, and the sporopollenin metabolon in maize anther remains unknown. Here, we identified two maize GMS genes, *ZmTKPR1‐1* and *ZmTKPR1‐2*, by CRISPR/Cas9 mutagenesis of 14 lipid metabolic genes with anther stage‐specific expression patterns. Among them, *tkpr1‐1/‐2* double mutants displayed complete male sterility with delayed tapetum degradation and abortive pollen. *ZmTKPR1‐1* and *ZmTKPR1‐2* encode tetraketide α‐pyrone reductases and have catalytic activities in reducing tetraketide α‐pyrone produced by ZmPKSB (polyketide synthase B). Several conserved catalytic sites (S128/130, Y164/166 and K168/170 in ZmTKPR1‐1/‐2) are essential for their enzymatic activities. Both *ZmTKPR1‐1* and *ZmTKPR1‐2* are directly activated by ZmMYB84, and their encoded proteins are localized in both the endoplasmic reticulum and nuclei. Based on protein structure prediction, molecular docking, site‐directed mutagenesis and biochemical assays, the sporopollenin biosynthetic metabolon ZmPKSB‐ZmTKPR1‐1/‐2 was identified to control pollen exine formation in maize anther. Although ZmTKPR1‐1/‐2 and ZmPKSB formed a protein complex, their mutants showed different, even opposite, defective phenotypes of anther cuticle and pollen exine. Our findings discover new maize GMS genes that can contribute to male‐sterility line‐assisted maize breeding and also provide new insights into the metabolon‐regulated sporopollenin biosynthesis in maize anther.

## Introduction

In flowering plants, the anther cuticle and pollen exine are two crucial lipid layers for pollen grain formation (Wan *et al*., 2020). The former covers the anther outer surface and protects pollen development from biotic and abiotic stresses during anthesis (Yeats and Rose, 2013). The latter, the outermost layer of the pollen wall, plays an important role in pollen–stigma interaction and double fertilization in addition to protecting pollen grains after releasing them from anthers (Shi *et al*., 2015). Since lipids and their derivatives are the major chemical components of both layers, they often display defective structures when lipid biosynthesis or transport pathways are blocked. Plant mutants with defective anther cuticle or pollen exine usually display male‐sterile or partially sterile phynotypes (Wan *et al*., 2020).

Mutation in a single nuclear gene may disrupt plant male reproduction, and such a gene is defined as a genic male‐sterility (GMS) gene (Wan *et al*., 2019). So far, more than 200 GMS genes have been identified in plants, a large proportion of which are lipid metabolic GMS genes, including 40 in *Arabidopsis*, 27 in rice and 12 in maize (Chen *et al*., 2022; Wan *et al*., 2019, 2020). These GMS genes largely contribute to the genetic network establishment of anther lipid metabolism in model plant species such as *Arabidopsis* and rice. However, the network remains unclear in maize due to the relatively fewer characterized lipid metabolic GMS genes. Of maize lipid metabolic GMS genes, nine are involved in lipid biosynthesis, such as *ZmMs20/IRREGULAR POLLEN EXINE1* (*ZmIPE1*) (Chen *et al*., 2017; Wang *et al*., 2019), *ZmMs25/ZmMs6021* (Tian *et al*., 2017; Zhang *et al*., 2021), *ZmMs26* (Djukanovic *et al*., 2013; Singh *et al*., 2017), *ZmMs30* (An *et al*., 2019), *ZmMs33* (Li *et al*., 2022, 2023; Xie *et al*., 2018; Zhang *et al*., 2018b; Zhu *et al*., 2019, 2020), *ZmMs45* (Cigan *et al*., 2001), *ABNORMAL POLLEN VACUOLATION1* (*ZmAPV1*) (Somaratne *et al*., 2017), *ZmIPE2* (Huo *et al*., 2020) and *POLYKETIDE SYNTHASE B* (*ZmPKSB*) (Liu *et al*., 2022). The rest encode lipid transfer proteins or ABCG transporters that are involved in lipid transport in maize anther (Fang *et al*., 2023; Wu *et al*., 2022), including *ZmMs2/ZmABCG26* (Choi *et al*., 2011; Jiang *et al*., 2021b; Xu *et al*., 2021), *ZmMs13/ZmABCG2a* (Fang *et al*., 2022) and *ZmMs44* (Fox *et al*., 2017). All mutants of these genes exhibit defective pollen exine and anther cuticle phenotypes. Nevertheless, their interaction relationships and whether they synergistically participate in a specific lipid metabolic pathway for pollen exine and anther cuticle formation are barely reported in maize.

Notably, most GMS genes in plants have been identified using the forward genetic strategy, which depends on the available GMS mutants, and the identification process is time‐consuming. Recently, eight GMS genes encoding transcription factors (TFs) have been successfully identified in maize via CRISPR/Cas9 genome editing, demonstrating its effectiveness in the discovery of GMS genes and mutant creation (Jiang *et al*., 2021a). Based on cytological observation and landmark developmental events, maize anther development is divided into 14 stages (S1–S14), and the characteristics of each stage are described in detail (Wan *et al*., 2019). Pollen exine formation begins with the primexine appearance at S8b in maize. At S9, a thin exine composed of continuous tectum, foot layer and bacula is formed, then thickened apparently at S10, and completed at S11 and S12 (An *et al*., 2019). Anther cuticle appears at S10, becomes apparent at S11 and matures at S12 and S13 (An *et al*., 2019). Correspondingly, most maize lipid metabolic GMS genes for anther and pollen development have expression peaks during stages S8–S10. Therefore, selecting candidate lipid metabolic genes with expression patterns from S8 to S10 for CRISPR/Cas9 mutagenesis may efficiently discover new GMS genes in maize.

Sporopollenin, a complex biopolymer of lipidic monomers and phenylpropanoid, is the main constituent of pollen exine (Li *et al*., 2019; Shi *et al*., 2015; Xue *et al*., 2020). In *Arabidopsis* and rice, sporopollenin biosynthesis depends on a lipid metabolon consisting of acyl‐CoA synthetase (ACOS), PKS and TKPR (Grienenberger *et al*., 2010; Lallemand *et al*., 2013; Yang *et al*., 2023). Mutations in any of these genes may result in complete or partial male fertility. These sporopollenin biosynthetic enzymes interact and use the products of upstream enzymes as substrates for sequential catalytic reactions to ensure metabolic efficiency (Lallemand *et al*., 2013; Yang *et al*., 2023). Although the molecular mechanisms of each component affecting pollen exine formation remain unknown, such a metabolon has also been reported in tobacco, moss, *Hypericum perforatum* and canola (Daku *et al*., 2016; Karppinen *et al*., 2008; Qin *et al*., 2016; Wang *et al*., 2013). This suggests that a conserved sporopollenin biosynthetic pathway is likely present in land plants. Recently, we have found that ZmPKSB is essential for normal male fertility in maize (Liu *et al*., 2022). However, whether the PKSB–TKPR sporopollenin metabolon exists in maize has not yet been uncovered.

Here, we found that *ZmTKPR1‐1* and *ZmTKPR1‐2* are GMS genes by screening and phenotyping 14 maize lipid metabolic gene mutants generated by CRISPR/Cas9. Among them, only *tkpr1‐1/‐2* double mutants display complete male sterility with delayed tapetum degradation and defective pollen exine and anther cuticles. Both *ZmTKPR1‐1* and *ZmTKPR1‐2* are directly activated by TF ZmMYB84. ZmTKPR1‐1 and ZmTKPR1‐2 are dual‐localized in the ER and nuclei. ZmPKSB, ZmTKPR1‐1 and ZmTKPR1‐2 interact with each other and form a complex, which constitutes a biosynthetic sporopollenin metabolon. The detailed functional differences between ZmTKPR1‐1/‐2 and ZmPKSB in lipid metabolism for anther cuticle and pollen exine formation are revealed.

## Results

### Expression patterns of 14 lipid metabolic genes during maize anther development and their CRISPR/Cas9‐induced mutations

**Figure 1:**
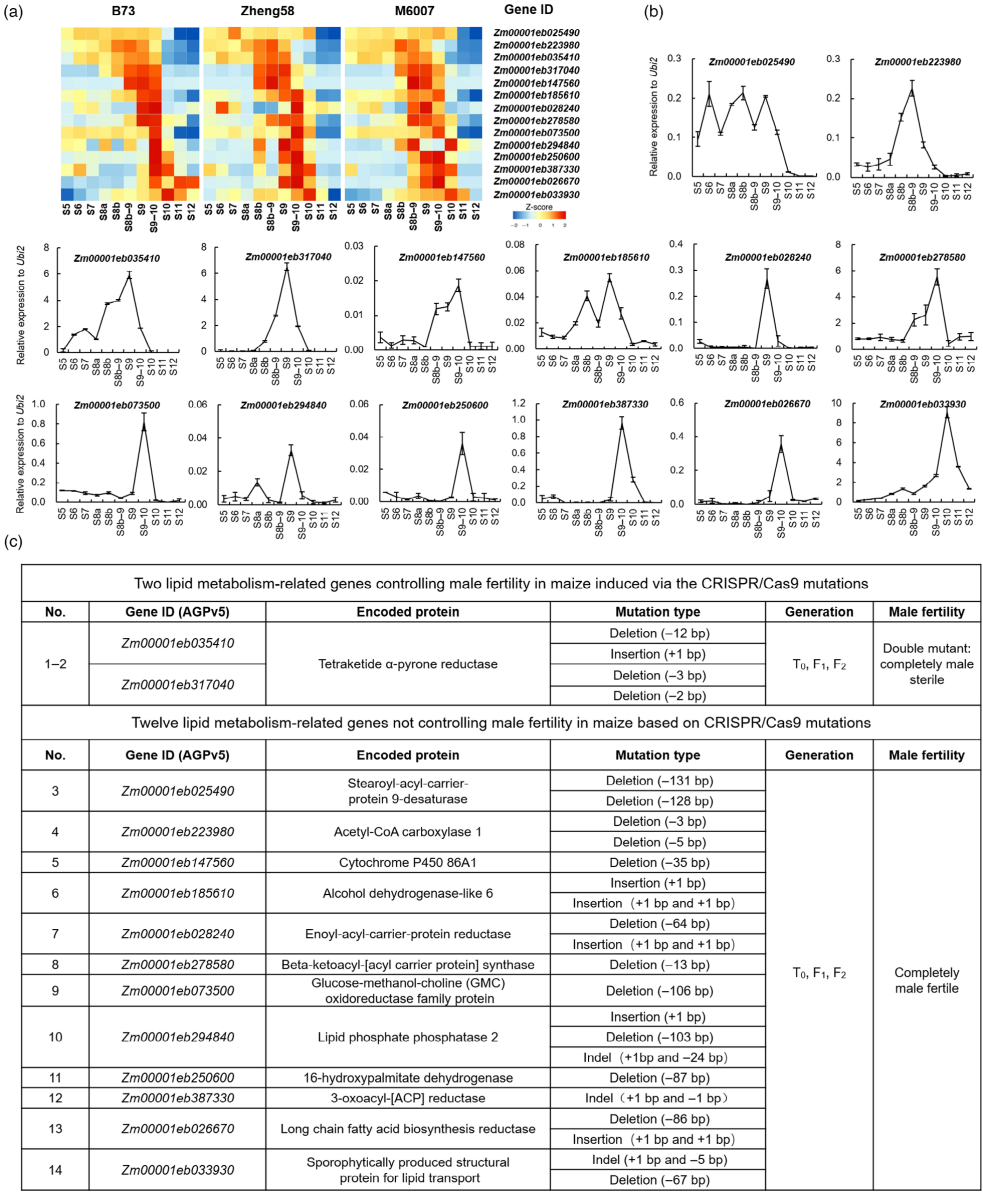
Expression patterns of 14 lipid metabolic genes during maize anther development and their CRISPR/Cas9‐induced mutations. (a) Expression patterns of 14 maize lipid metabolic genes during anther developmental stages S5 to S12 based on anther transcriptome analysis in B73, Zheng58 and M6007 genetic backgrounds. (b) qRT‐PCR analyses of the 14 maize lipid metabolic genes during B73 anther developmental stages S5 to S12. (c) Gene information, CRISPR/Cas9‐induced mutation types and their corresponding male‐sterility or ‐fertility phenotypes of the 14 investigated maize lipid metabolic genes.

Given that most lipid metabolic GMS genes for pollen exine formation are highly expressed during anther developmental stages S8–S10, we first selected 14 maize metabolic genes with expression peaks at these stages based on transcriptome analysis of anther RNA‐Seq data in three maize inbred lines (B73, Zheng58 and M6007) and then verified their expression patterns by qRT‐PCR analysis (Figure 1a,b; Table S1). Among them, *Zm00001eb025490* exhibited high expression levels from S5 to S9–10, with three expression peaks at S6, S8b and S9, respectively. *Zm00001eb223980* displayed high expression levels from S8b to S9, with a peak at S8b–9. *Zm00001eb035410*, *Zm00001eb317040*, *Zm00001eb028240* and *Zm00001eb294840* shared similar expression patterns, with the common peaks at S9. *Zm00001eb185610* exhibited two expression peaks at S8b and S9, respectively. *Zm00001eb147560* was highly expressed from S8b–9 to S9–10, with a peak at S9–10. *Zm00001eb073500*, *Zm00001eb250600*, *Zm00001eb387330* and *Zm00001eb026670* had similar expression patterns, with the common peaks at S9–10. *Zm00001eb033930* was expressed at the late stages of anther development, with a peak at S10 (Figure 1a,b). Thus, the temporal expression patterns of these 14 genes covered the critical periods of exine formation in maize anther.

**Figure 2:**
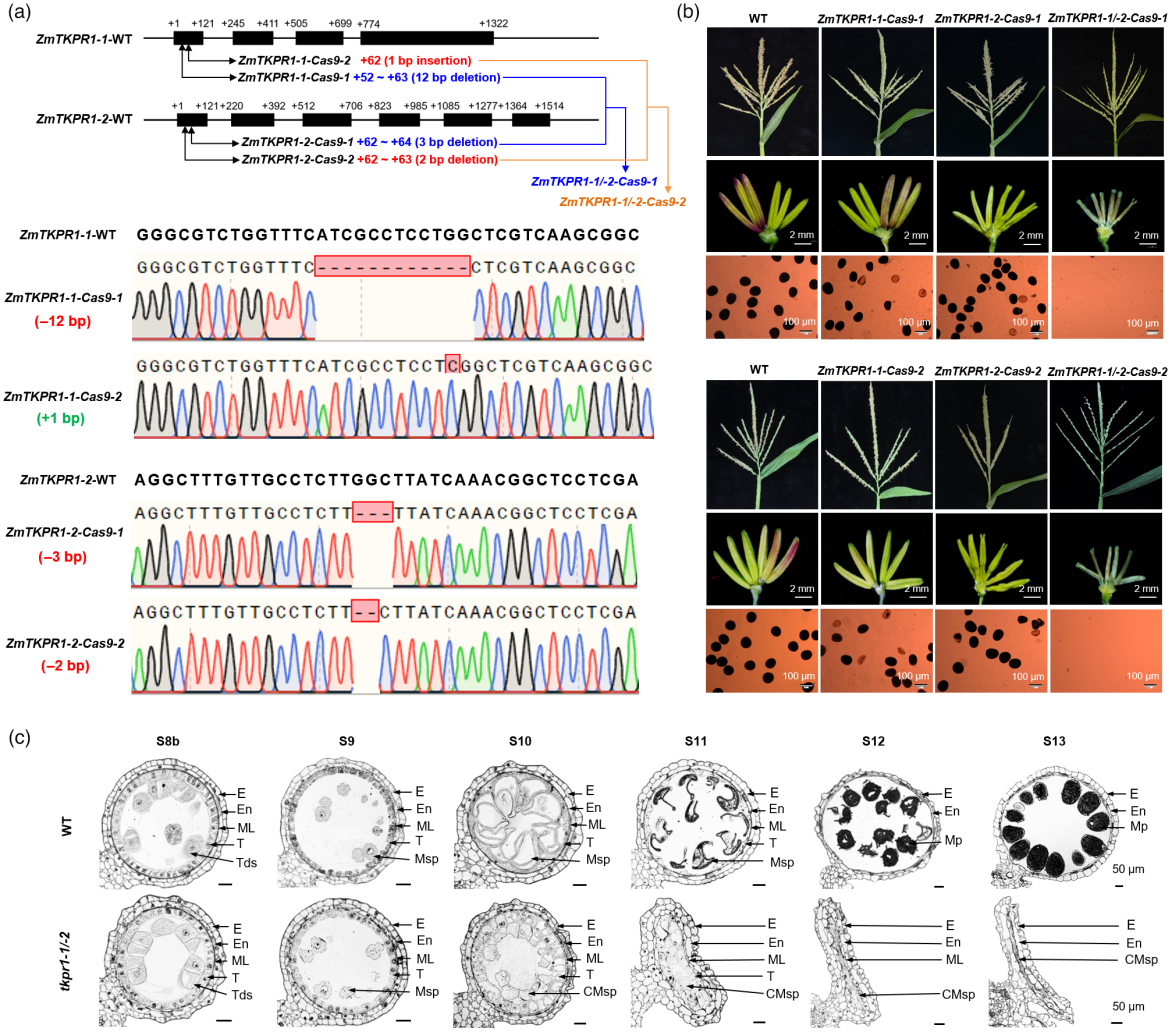
CRISPR/Cas9 mutagenesis and the derived mutant phenotypic characterization of *ZmTKPR1‐1/‐2*. (a) Gene structure and mutation analysis of *ZmTKPR1‐1/‐2* in WT and the single knockout lines (*ZmTKPR1‐1‐Cas9‐1*, *‐2*, *ZmTKPR1‐2‐Cas9‐1* and *‐2*) and double knockout lines (*ZmTKPR1‐1/‐2‐Cas9‐1* and *ZmTKPR1‐1/‐2‐Cas9‐2*) generated by the CRISPR/Cas9 genome editing. (b) Comparison of tassels, anthers and pollen grains stained with I_2_‐KI among WT, the four single‐gene knockout lines and two double knockout lines. (c) Transverse sections of WT and *tkpr1‐1/‐*2 anthers from stages S8b to S13. CMsp, collapsed microspore; E, epidermis; En, endothecium; ML, middle layer; Mp, mature pollen; Msp, microspore; T, tapetum; Tds, tetrads.

We then investigated their potential functions in maize anther and pollen development through CRISPR/Cas9‐induced gene mutagenesis (Figure S1). Consequently, two of them, *Zm00001eb035410* and *Zm00001eb317040*, were found to be essential for maize male fertility, and their double mutants, not single mutants, showed complete male sterility. Both genes are homologues and encode tetraketide α‐pyrone reductases, ZmTKPR1‐1 and ZmTKPR1‐2, respectively (Figures 1c and 2). However, single mutants of the remaining 12 genes showed normal male fertility in maize, including 11 lipid biosynthetic genes (*Zm00001eb025490*, *Zm00001eb223980*, *Zm00001eb147560*, *Zm00001eb185610*, *Zm00001eb028240*, *Zm00001eb278580*, *Zm00001eb073500*, *Zm00001eb294840*, *Zm00001eb250600*, *Zm00001eb387330* and *Zm00001eb026670*) and one lipid transport‐related gene (*Zm00001eb033930*) (Figures 1c, S2a,b and S3). These results indicate that CRISPR/Cas9‐based genome editing combined with anther transcriptome analysis is an effective approach to discovering new GMS genes, especially for those depending on simultaneous mutations of mutilple homologous genes.

### *tkpr1‐1/‐2* double mutants exhibit defective pollen exine and anther cuticle

Both *ZmTKPR1‐1* and *ZmTKPR1‐2* are orthologues of Os*TKPR1* in rice and *AtTKPR1* in *Arabidopsis* (Grienenberger *et al*., 2010; Xu *et al*., 2019). To explore their possible functions in maize, we generated Cas9‐free homozygous single‐ and double‐gene knockout lines of *ZmTKPR1‐1*/*‐2* with T_0_, F_1_ and F_2_ generations (Figures 1c and 2a) according to the method previously reported (Jiang *et al*., 2021a).

Deletion or insertion mutations in four single‐gene knockout lines (*ZmTKPR1‐1‐Cas9‐1/‐2* and *ZmTKPR1‐2‐Cas9‐1/‐2*) and two double‐gene mutants (*ZmTKPR1‐1/‐2‐Cas9‐1* and *ZmTKPR1‐1/‐2‐Cas9‐2*) resulted in an open reading frame shift or loss of critical amino acids in ZmTKPR1‐1 and ZmTKPR1‐2 (Figure 2a). All single‐gene knockout lines of either *ZmTKPR1‐1* or *ZmTKPR1‐2* displayed slightly partial male sterility with few abortive pollen grains (Figure S2c). However, both double‐gene knockout lines of *ZmTKPR1‐1/‐2* exhibited complete male sterility with smaller anthers and no visible pollen grains compared with WT (Figure 2b). To perform detailed phenotypic identification, we used one double mutant *ZmTKPR1‐1/‐2‐Cas9‐1* (named *tkpr1‐1/‐2*) for further study.

To investigate the cytological defects in *tkpr1‐1/‐2* anthers, we observed transverse sections of WT and *tkpr1‐1/‐2* anthers from stages S5–S13. Anther and microspore development were comparable between WT and *tkpr1‐1/‐2* until S9 (Figures 2c and S4a). From S10 to S13, WT microspores underwent vacuolization, gradually accumulated starch, and eventually formed mature pollen grains. However, *tkpr1‐1/‐2* microspores were abnormal in terms of shape and size, failed to accumulate starch and eventually collapsed (Figure 2c). The WT tapetum initiated degradation at S10 and was completely degraded at S11, while the *tkpr1‐1/‐2* tapetum thickened at S10 and was distinctly visible at S11 (Figure 2c).

Scanning electron microscopy (SEM) showed no significant difference in anther outer and inner surfaces, and microspores between WT and *tkpr1‐1/‐2* before stage S9 (Figure S4b). From S10 to S13, the outer and inner surfaces of *tkpr1‐1/‐2* anther were smooth, and no three‐dimensional knitting cuticle and Ubisch bodies appeared when compared with those of WT. Additionally, *tkpr1‐1/‐2* microspores adhered to each other and displayed defective vacuolization and starch accumulation (Figure S4b). These results indicate that simultaneous mutations of *ZmTKPR1‐1/‐2* result in the abnormal development of anther cuticle, Ubisch body and pollen grains in maize.

**Figure 3:**
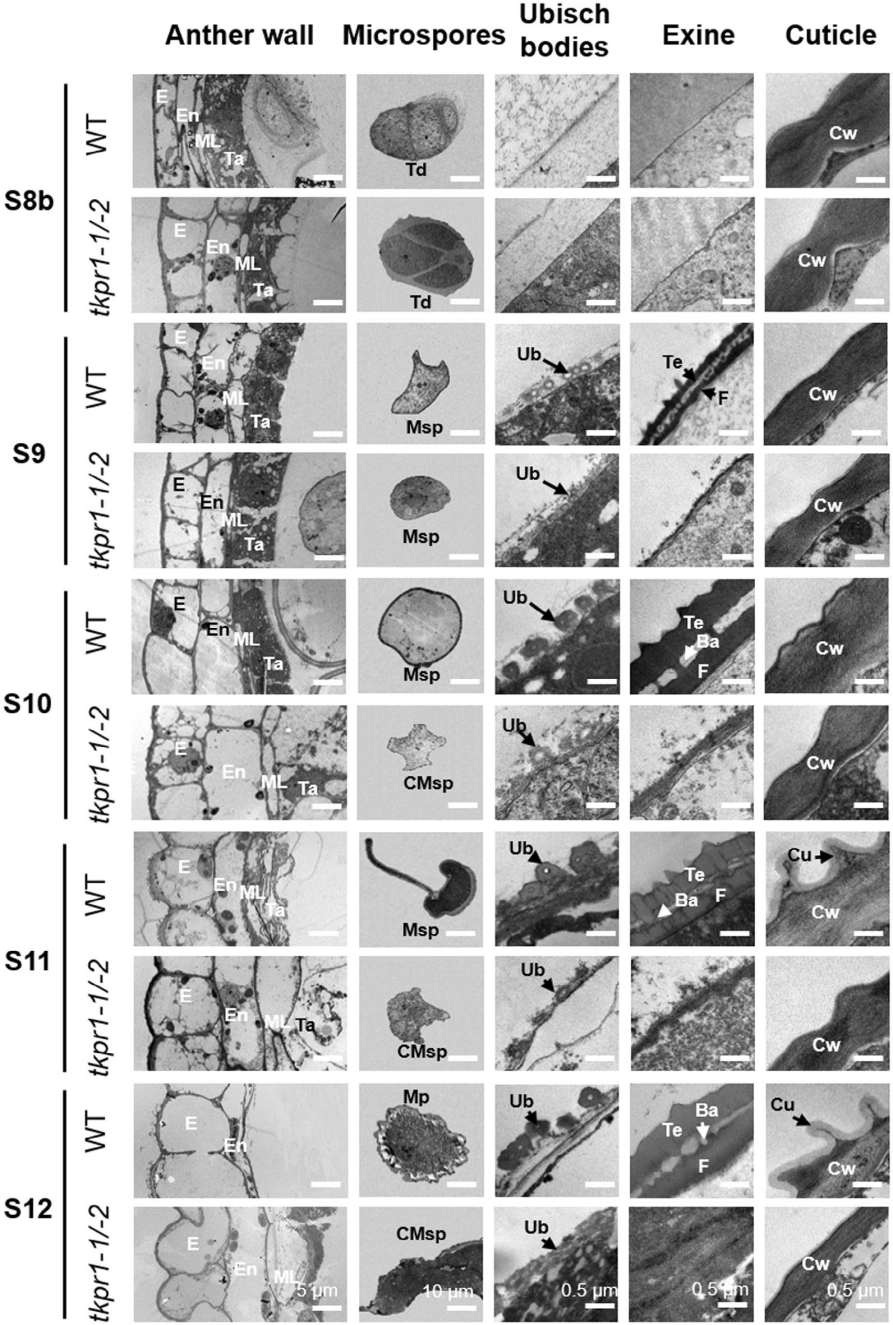
TEM observation of WT and *tkpr1‐1/‐2* anthers from stages S8b to S12. Ba, baculua; CMsp, collapsed microspore; Cu, cuticle; Cw, cell wall; E, epidermis; En, endothecium; F, foot layer; ML, middle layer; Mp, mature pollen; Msp, microspore; Ta, tapetum; Td, tetrad; Te, tectum; Ub, Ubisch body.

Consistent with the transverse section and SEM observations, no distinguishable difference in anther and microspore development between *tkpr1‐1/‐2* and WT was observed by transmission electron microscopy (TEM) analysis at stage S8b (Figure 3). Developmental differences were observed from S9 to S12, during which *tkpr1‐1/‐2* anther exhibited inflated tapetum, delayed tapetal degeneration, collapsed microspores, unformed Ubisch bodies and pollen exine and disappeared three‐dimensional knitting cuticle compared with WT anther (Figures 3 and S4b). Furthermore, TUNEL assays revealed that tapetal programmed cell death (PCD) was delayed in *tkpr1‐1/‐2* anther (at S11 and S12) compared to WT anther (at S10) (Figure S4c), indicating that *ZmTKPR1‐1* and *‐2* are essential for the timely tapetal PCD and degeneration. Taken together, the *tkpr1‐1/‐2* double mutations lead to delayed tapetal degeneration; a lack of Ubisch bodies and pollen exine; three‐dimensional knitting anther cuticle; and ultimately complete male sterility.

### Molecular characterization and transcriptional regulation of *ZmTKPR1‐1/‐2*

**Figure 4:**
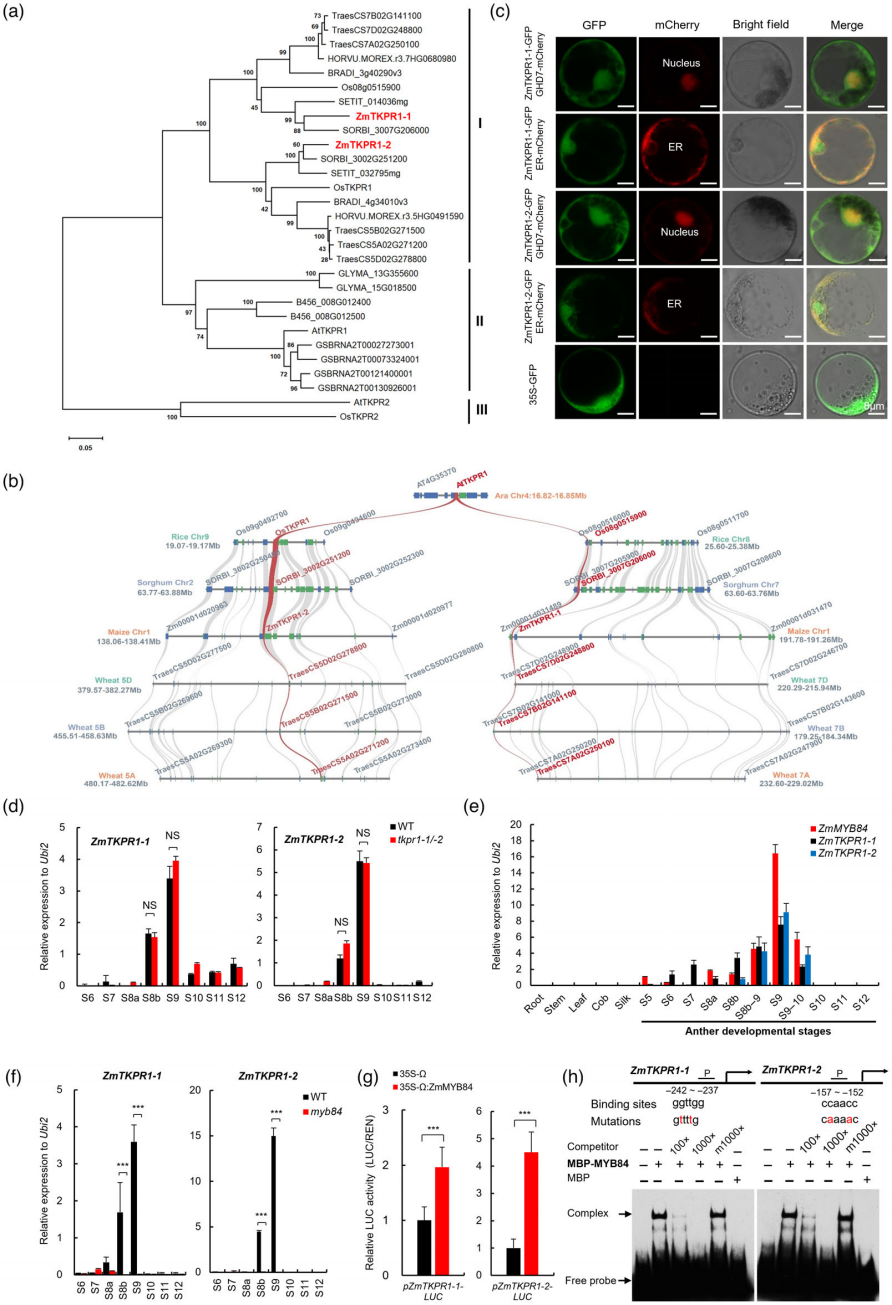
Molecular characterization and transcriptional regulation analyses of *ZmTKPR1‐1* and *ZmTKPR1‐2*. (a) Phylogenetic analysis of ZmTKPR1‐1, ZmTKPR1‐2 and their orthologues in 11 plant species. The analysis included 29 amino acid sequences from *Arabidopsis thaliana* (At), *Bradchypodium distachyon* (Bd), *Brassica napus* (Bn), *Glycine max* (Gm), *Gossypium raimondii* (Gr), *Hordeum vulgare* (Hv), *Oryza sativa* (Os), *Setaria italica* (Si), *Sorghum bicolor* (Sb), *Triticum aestivum* (Ta) and *Zea mays* (Zm). The numbers on the branches represent the bootstrap values of the phylogenetic tree. (b) Microsynteny analysis of *ZmTKPR1‐1* and *ZmTKPR1‐2* loci and their orthologues from four monocots and one eudicot. Genes shown in red indicate *ZmTKPR1‐1*, *ZmTKPR1‐2* and their orthologues. Genes flanking *ZmTKPR1‐1*, *ZmTKPR1‐2* and their orthologues are shown in grey. The abbreviations of At, Os, Sb, Ta and Zm are the same as these in (a). (c) Subcellular localization of ZmTKPR1‐1 and ZmTKPR1‐2 in maize protoplasts. The ZmTKPR1‐1‐GFP and ZmTKPR1‐2‐GFP were co‐transformed with the ER‐mCherry and Nucleus‐mCherry as ER and nucleus markers, respectively. The 35S‐GFP vector was used as a negative control. (d) qPCR analysis of *ZmTKPR1‐1* and *ZmTKPR1‐2* in WT and *tkpr1‐1/‐2* anthers. (e) Spatiotemporal expression of *ZmTKPR1‐1*, *ZmTKPR1‐2* and *ZmMYB84* detected by qPCR analysis. (f) qPCR analysis of *ZmTKPR1‐1* and *ZmTKPR1‐2* in WT and *myb84* anthers. (g) Transient dual‐luciferase reporter (TDLR) assay of *ZmTKPR1‐1* and *ZmTKPR1‐2* promoter activities activated by ZmMYB84 in maize protoplasts. Data represent mean ± SD, *n* = 3. (h) EMSA showing ZmMYB84 binding to the ggttgg or ccaacc motif in *ZmTKPR1‐1* and *ZmTKPR1‐2* promoters *in vitro*. For (d, f and g), NS and *** indicate the significant levels of *P* > 0.05 and *P* < 0.001 determined by a two‐tailed Student's *t*‐test, respectively.

To investigate the evolutionary history of ZmTKPR1‐1/‐2, we conducted a protein sequence alignment with their orthologues from 11 different plant species. Results showed that the putative common NAD(P)H binding domain and NAD(P)‐dependent epimerase/ dehydratase domain are conserved across these species (Figure S5). Phylogenetic analysis classifies these orthologues into three clades. Clade I contains ZmTKPR1‐1, ZmTKPR1‐2 and their monocot orthologues, including rice OsTKPR1 (Os09g0493500) (the closest paralogue of ZmTKPR1‐2) (Xu *et al*., 2019) and Os08g0515900 (the closest paralogue of ZmTKPR1‐1), while all the members of Clade II are from dicots, including *Arabidopsis* AtTKPR1 (Tang *et al*., 2009). AtTKPR2 and OsTKPR2 are distributed in Clade III, which is separated from Clade I and II branches, indicating that they have a far phylogenetic relationship with ZmTKPR1‐1 and ‐2 (Figure 4a). Microsynteny analysis revealed that ZmTKPR1‐1 and ‐2 are probably duplicated paralogues after evolutionary differentiation of monocots and dicots, as their orthologues in monocots rice, sorghum and wheat showed good syntenies with the AtTKPR1 single locus (Figure 4b). These results suggest that ZmTKPR1‐1 and ‐2 and their orthologues were relatively conserved during the evolution of gramineous plants.

Subcellular localization analysis performed in maize protoplasts and tobacco leaves revealed that both ZmTKPR1‐1 and ‐2 were dual‐localized in the nuclei and ER (Figures 4c and S6a,b). qPCR analysis in different maize tissues showed that *ZmTKPR1‐1* and *‐2* transcripts were detected exclusively in anthers at specific developmental stages and both peaked at S9 (Figures 4d,e and S6c,d). Remarkably, no significant difference was detected in the expression levels of *ZmTKPR1‐1* and *‐2* between *tkpr1‐1/‐2* and WT anthers, suggesting that the male‐sterility phenotype of *tkpr1‐1/‐2* results from their protein function defects.

Given that TF ZmMYB84 is required for tapetal PCD and pollen exine formation (Fang *et al*., 2022; Jiang *et al*., 2021a) and the expression pattern of *ZmMYB84* overlaps with those of *ZmTKPR1‐1* and *‐2* from S8a to S9–10, with the same peak at S9 (Figures 4e and S6d), we speculate that *ZmTKPR1‐1* and *‐2* may be regulated by ZmMYB84. qPCR analysis revealed that *ZmTKPR1‐1* and *‐2* transcripts were almost undetectable in *myb84* anther (Figures 4f and S6e). Thus, ZmMYB84 is necessary for the expression of *ZmTKPR1‐1* and *‐2*. To further confirm whether *ZmTKPR1‐1* and *‐2* are the direct target genes of ZmMYB84, we conducted a transient dual‐luciferase reporter (TDLR) assay and an electrophoretic mobility shift assay (EMSA) and found that ZmMYB84 bound to the promoters of *ZmTKPR1‐1* and *‐2* and activated their promoter activities (Figure 4g,h). Collectively, ZmMYB84 directly activates *ZmTKPR1‐1* and *ZmTKPR1‐2* expression.

### ZmPKSB, ZmTKPR1‐1 and ZmTKPR1‐2 interact and form a multienzyme complex

Growing evidence has proved that various proteins involved in lipid biosynthetic pathways can form dynamic protein–protein and protein‐lipid interactomes or metabolons (Nakamura, 2017). Since ZmPKSB (Liu *et al*., 2022), ZmTKPR1‐1 and ‐2 exhibit similar expression patterns during anther development and their encoded proteins are localized in the ER (Figures 4c–e and S6), we speculated that ZmPKSB, ZmTKPR1‐1 and ‐2 may interact and form a complex to conduct their functions in maize anther development.

**Figure 5:**
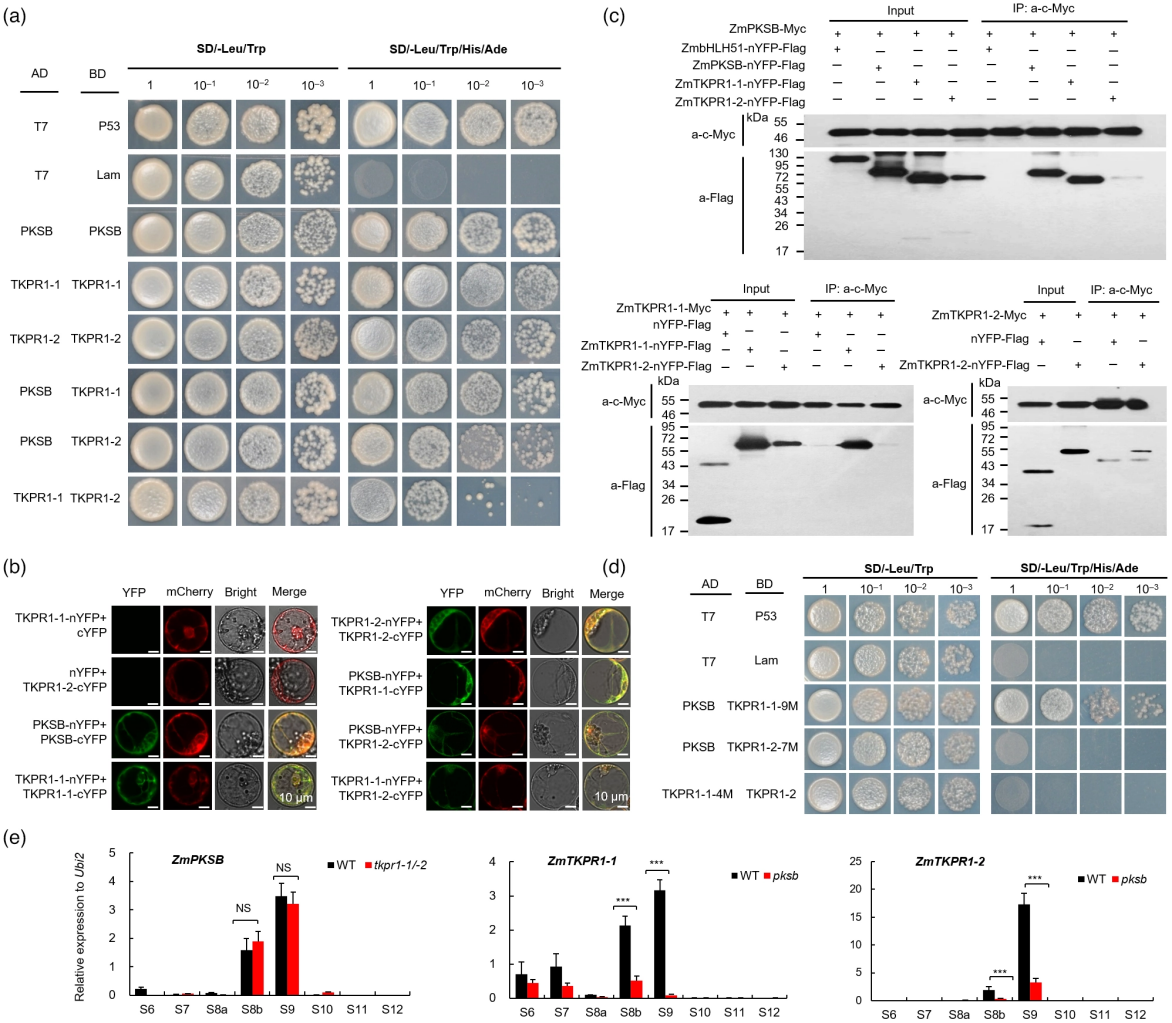
Interactions of ZmPKSB, ZmTKPR1‐1 and ZmTKPR1‐2. (a) Protein interactions of ZmPKSB, ZmTKPR1‐1 and ZmTKPR1‐2 shown by the Y2H assay. (b) BiFC analysis demonstrates the interactions of ZmPKSB, ZmTKPR1‐1 and ZmTKPR1‐2 in maize protoplasts. Protein name‐n and ‐c indicate nYFP and cYFP fusions, respectively. HDEl‐mCherry was used as an ER marker. (c) Protein interactions shown by the Co‐IP assay in maize protoplasts. The nYFP‐FLAG and ZmbHLH51 were used as negative controls. (d) Protein interactions of amino acid substitution variants that cannot form hydrogen bonds are shown by the Y2H assay. TKPR1‐1‐9M, TKPR1‐2‐7M and TKPR1‐1‐4M indicate nine, seven and four amino acid substitution variants, respectively. (e) qPCR analysis of *ZmPKSB* in WT and *tkpr1‐1/‐2* anthers and *ZmTKPR1‐1* and *ZmTKPR1‐2* in WT and *pksb* anthers. NS and *** indicate the significant levels of *P* > 0.05 and *P* < 0.001 determined by a two‐tailed Student's *t*‐test, respectively. For (a and d), T7/P53 and T7/Lam were used as positive and negative controls, respectively. SD‐Trp‐Leu indicates double dropout medium; SD‐Trp‐Leu‐His‐Ade indicates quadruple dropout medium.

To test this hypothesis, we first predicted pairwise interactions between these three proteins using AlphaFold2. Results showed that ZmPKSB, ZmTKPR1‐1 and ‐2 could form both homodimers and heterodimers (ZmPKSB/ZmTKPR1‐1, ZmPKSB/ZmTKPR1‐2 and ZmTKPR1‐1/ZmTKPR1‐2), and that the high zdock scores indicated good molecular dockings (Figure S7a–d). Next, we performed yeast two‐hybrid (Y2H), bimolecular fluorescence complementation (BiFC) and co‐immunoprecipitation (Co‐IP) assays to validate these protein–protein interactions (PPIs). These results thus obtained indicated that ZmPKSB, ZmTKPR1‐1 and ‐2 interact and form a multienzymatic complex (Figure 5a–c).

Intermolecular hydrogen bonds play crucial roles in the structural organization and stabilization of a protein complex (Fernandez and Scheraga, 2003). Based on the molecular docking results of PPIs, nine, seven and four intermolecular hydrogen bonds on the interfaces of three heterodimers (ZmPKSB/ZmTKPR1‐1, ZmPKSB/ZmTKPR1‐2 and ZmTKPR1‐1/ZmTKPR1‐2) were predicted, respectively. Next, we constructed three variants with amino acid substitutions, ZmTKPR1‐1‐9M, ZmTKPR1‐2‐7M and ZmTKPR1‐1‐4M, to validate these binding sites. Y2H assay results showed that ZmPKSB and ZmTKPR1‐2‐7M as well as ZmTKPR1‐1‐4M and ZmTKPR1‐2 failed to interact, indicating these binding sites were required for their protein interactions (Figure 5d).

qPCR analysis showed that *pksb* mutation significantly reduced *ZmTKPR1‐1* and *‐2* transcripts, but *ZmPKSB* transcript was not affected by *tkpr1‐1/‐2* mutations (Figures 5e and S7e), suggesting that *ZmPKSB* may act upstream of *ZmTKPR1‐1* and *‐2* with a possible sequential enzymatic reaction of ZmPKSB‐ZmTKPR1‐1/‐2.

### ZmPKSB, ZmTKPR1‐1 and ZmTKPR1‐2 enzymatic activities define a sporopollenin metabolon in maize

In *Arabidopsis* and rice, PKS and TKPR are involved in a conserved sporopollenin metabolon (Lallemand *et al*., 2013; Yang *et al*., 2023). Mutations of both *ZmPKSB* and *ZmTKPR1‐1/‐2* lead to male sterility, and ZmPKSB and ZmTKPR‐1‐1/‐2 form a multienzyme complex, implying that a similar metabolon may also exist in maize anther.

**Figure 6:**
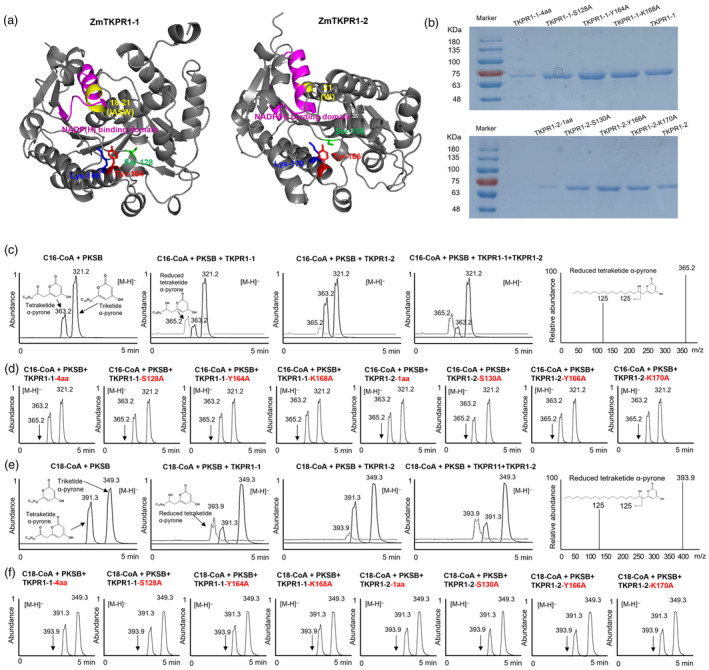
Enzyme activity analysis of the recombinant ZmPKSB, ZmTKPR1‐1 and ZmTKPR1‐2 proteins. (a) The simulated three‐dimensional structure of ZmTKPR1‐1 and ZmTKPR1‐2. The NADP binding domain is shown in purple; serine (Ser), tyrosine (Tyr) and lysine (Lys) are shown in green, red and blue, respectively; and amino acid deletions in generated variant proteins are shown in yellow. (b) SDS‐PAGE of the MBP‐tagged proteins TKPR1‐1, TKPR1‐2, six single amino acid substitution variants (S128A, Y164A, K168A, S130A, Y166A and K170A) and two deletion variants (−4 and −1aa) purified from *E. coli*. (c) HPLC analysis of the products of enzymatic reactions catalysed by PKSB‐MBP, TKPR1‐1‐MBP and TKPR1‐2‐MBP with C16‐CoA + malonyl‐CoA as substrates. The cognate triketide, tetraketide α‐Pyrone and reduced tetraketide α‐pyrone reaction products were detected. The m/z value of the [M‐H]‐ion of each product is indicated. (d) HPLC analysis of the products of enzymatic reactions catalysed by six single amino acid substitution variants (S128A, Y164A, K168A, S130A, Y166A and K170A) and two deletion variants (−4 and −1aa) with C16‐CoA + malonyl‐CoA as substrates. (e) HPLC analysis of the products of enzymatic reactions catalysed by PKSB‐MBP, TKPR1‐1‐MBP and TKPR1‐2‐MBP with C18‐CoA + malonyl‐CoA as substrates. (f) HPLC analysis of the products of enzymatic reactions catalysed by six single amino acid substitution variants (S128A, Y164A, K168A, S130A, Y166A and K170A) and two deletion variants (−4 and −1aa) with C18‐CoA + malonyl‐CoA as substrates.

In addition to PPI, an important feature of metabolon is that a multienzyme complex can afford highly efficient sequential catalytic reactions (Zhang and Fernie, 2021). To test whether this feature occurs in the ZmPKSB‐ZmTKPR1‐1/‐2 complex, we performed a series of enzyme activity experiments using purified recombinant proteins of PKSB‐MBP (Liu *et al*., 2022), ZmTKPR1‐1‐MBP and ZmTKPR1‐2‐MBP, and eight variants of ZmTKPR1‐1/‐2 from *E. coli* (Figure 6a,b). These variants include two versions with amino acid deletion (TKPR1‐1‐4aa and TKPR1‐2‐1aa, where mutation sites correspond to those in *tkpr1‐1/‐2* mutant) and six variants with single amino acid substitutions (TKPR1‐1: S128A, Y164A and K168A, and TKPR1‐2: S130A, Y166A and K170A, where mutation sites are from predicted catalytic sites by homology modelling).

Our previous study has shown that ZmPKSB condenses malonyl‐CoA, C16:0‐CoA and C18:0‐CoA to generate tri‐ and tetraketide α‐pyrone (Liu *et al*., 2022). In this study, with C16:0‐CoA and C18:0‐CoA as primary substrates and malonyl‐CoA as an extender molecule, we found that when two or three recombinant proteins (PKSB + TKPR1‐1, PKSB + TKPR1‐2 or PKSB + TKPR1‐1 + TKPR1‐2) were added together, the reduced tetraketide α‐pyrones were produced (Figure 6c,e). Notably, ZmTKPR1‐1 exhibited greater reductase activity towards tetraketide α‐pyrone than ZmTKPR1‐2, and the presence of both ZmTKPR1‐1 and ‐2 obviously increased the catalytic activities. In contrast, the eight variants of ZmTKPR1‐1 and ‐2 failed to produce reduced tetraketide α‐pyrone compounds, indicating that these conserved amino acid sites in the NAD‐dependent epimerase/dehydratase domain are essential for their enzymatic activities (Figure 6d,f).

Taken together, ZmPKSB, together with ZmTKPR1‐1 and ‐2, forms a conserved sporopollenin metabolon in maize anther, which facilitates the generation of the reduced tetraketide α‐pyrone, a sporopollenin precursor.

### *ZmPKSB* and *ZmTKPR1‐1/‐2* exhibit different effects on pollen exine and anther cuticle formation

**Figure 7:**
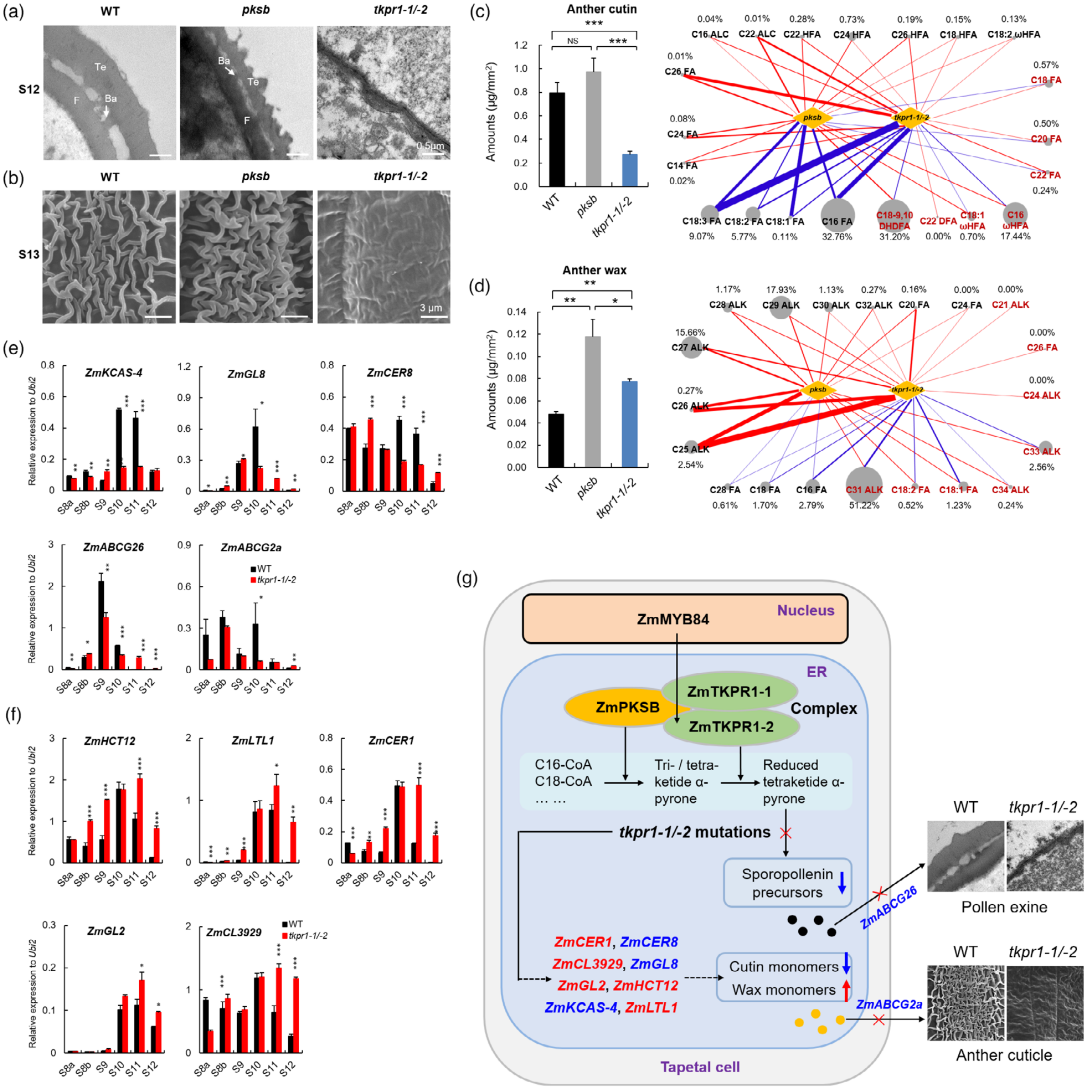
Cytological and lipid comparison among WT, *pksb* and *tkpr1‐1/‐2* anthers and ZmMYB84‐regulated ZmPKSB‐ZmTKPR1 metabolon affecting anther cuticle and pollen exine formation in maize. (a) TEM observation of pollen exine in WT, *pksb* and *tkpr1‐1/‐2* at stage S12. (b) SEM observation of anther outer surface in WT, *pksb* and *tkpr1‐1/‐2* at stage S13. (c) The amount of anther cutin in WT, *pksb* and *tkpr1‐1/‐2* at stage S13. (d) The amount of anther wax in WT, *pksb* and *tkpr1‐1/‐2* at stage S13. (e) qPCR analysis of five downregulated genes in *tkpr1‐1/‐2* anthers from stages S8a to S12. (f) qPCR analysis of five upregulated genes in *tkpr1‐1/‐2* anthers from stages S8a to S12. (g) A working model of the ZmMYB84‐regulated ZmPKSB‐ZmTKPR1 metabolon affecting anther cuticle and pollen exine formation in maize. Ba, baculua; F, foot layer; Te, tectum. For (c to f), *, ** and *** indicate the significant levels of *P* < 0.05, 0.01 and 0.001 determined by a two‐tailed Student's *t*‐test, respectively. For (c and d), the red or blue lines represent the increase or decrease of the cutin and wax monomers in mutant anthers, respectively. The thickness of the lines represents the change in magnitude in terms of increase or decrease.

Considering that ZmPKSB, ZmTKPR1‐1 and ‐2 are the components of sporopollenin metabolon mainly for pollen exine formation in maize and the formation processes of pollen exine and anther cuticle share certain common pathways of lipid metabolism (Wan *et al*., 2020), we performed cytological comparison among WT, *pksb* and *tkpr1‐1/‐2* mutants to determine whether *ZmPKSB* and *ZmTKPR1‐1/‐2* have comparable effects on the formation of both lipid layers. As expected, both mutants exhibited a thinner pollen exine compared to WT (Figure 7a). Nevertheless, the pollen exine of *tkpr1‐1/‐2* was obviously thinner than that of *pksb*. Additionally, anther cuticle showed opposite changes in both mutants. Compared with WT, anther cuticle was smooth in *tkpr1‐1/‐2* anther, but denser in *pksb* anther (Figure 7b), suggesting that *ZmPKSB* and *ZmTKPR1‐1/‐2* have different effects on pollen exine and anther cuticle formation.

Next, we analysed the contents of cutin, wax and total internal lipid in the anther cuticle, along with their constituents and monomers at stage S13, by GC–MS. The total anther cutin content in *tkpr1‐1/‐2* (0.272 μg/mm^2^) was significantly lower than that in WT (0.793 μg/mm^2^) and *pksb* (0.975 μg/mm^2^) (Figure 7c). These differences were primarily due to alterations in the amounts of 21 cutin monomers (Figures 7c and S8a; Table S2). Notably, the contents of C18–9, 10 DHDFA, C16 ωHFA and C18:1 ωHFA (accounting for 49.34% of the total cutin) were significantly decreased in *tkpr1‐1/‐2* anther but increased in *pksb* anther (Figure 7c). The total wax contents in both *tkpr1‐1/‐2* and *pksb* anthers (0.077 and 0.118 μg/mm^2^, respectively) were significantly higher than that (0.048 μg/mm^2^) in WT anthers. Moreover, the difference between *tkpr1‐1/‐2* and *pksb* was statistically significant (Figures 7d and S8b; Table S2), mainly due to the opposite alterations in contents of C31, C33 and C34 ALKs as well as C18:1 and C18:2 FAs (accounting for 55.77% of the total wax content) in the two mutant anthers (Figure 7d). Additionally, a significant difference in the total internal lipid content was found between *tkpr1‐1/‐2* (8.97 μg/mg DW) and *pksb* anthers (13.09 μg/mg DW) (Figure S8c; Table S2). These results are consistent with the different anther cuticle phenotypes displayed in *tkpr1‐1/‐2* and *pksb* (Figure 7b). Thus, *ZmPKSB* and *ZmTKPR1‐1/‐2* exhibit different effects on lipid metabolism for anther cuticle formation.

The abnormally thin pollen exine and smooth anther cuticle in *tkpr1‐1/‐2* anther suggest that sporopollenin and cutin/wax metabolic pathways may be disrupted by *tkpr1‐1/‐2* mutations. To confirm this, we measured expression alterations of one sporopollenin transport gene and nine cutin/wax‐related genes between WT and *tkpr1‐1/‐2* anthers from stages S8a to S12 by qPCR analysis. Results showed that the expression of the sporopollenin transport GMS gene *ZmABCG26* peaked at S9 in WT anther while being significantly reduced in *tkpr1‐1/‐2* anther (Figure 7e). Among these nine cutin/wax‐related genes, *ZmKCAS‐4*, *ZmGL8*, *ZmCER8* and *ZmABCG2a* were significantly downregulated in *tkpr1‐1/‐2* anther at S10, while *ZmHCT12*, *ZmLTL1*, *ZmCER1*, *ZmGL2* and *ZmCL3929* were significantly upregulated at S11 and S12 (Figure 7e,f). Collectively, *ZmTKPR1‐1* and *‐2* are required for pollen exine and anther cuticle formation, as reflected by the expression changes of sporopollenin‐ and cutin/wax‐related genes.

Our previous research has shown that the expression of *ZmPKSB* depends on ZmMYB84 (Liu *et al*., 2022). Taken together with these results obtained here, we proposed a working model to elucidate the roles of the ZmPKSB‐ZmTKPR1‐1/‐2 metabolon in maize anther tapetal cells (Figure 7g). *ZmPKSB*, *ZmTKPR1‐1* and *‐2* are activated by ZmMYB84, and the multienzyme complex ZmPKSB‐ZmTKPR1‐1/‐2 forms a sporopollenin metabolon in anther tapetal cells. Then, sequential enzymatic reactions occur to generate reduced tetraketide α‐pyrone by this metabolon. Subsequently, as crucial precursors of sporopollenin and cutin/wax, lipid metabolic products are transported by ZmABCG26 and ZmABCG2a to form pollen exine and anther cuticle. In *tkpr1‐1/‐2* anther, the reduced expression of ZmABCG26 blocks the transport of sporopollenin precursors, and the biosynthesis and transport of cutin/wax monomers are disturbed, likely due to the expression alterations of cutin/wax‐related genes, ultimately resulting in a greatly thin pollen exine and smooth anther cuticle.

## Discussion

### Integration of transcriptome, bioinformation and gene editing to discover new GMS genes in maize

In flowering plants, the anther cuticle is composed of lipidic polyester cutin and cuticular wax and pollen exine is mainly composed of sporopollenin and tryphine. All these materials are built by lipids or lipid derivatives, and many lipid metabolic GMS genes are indispensable for anther and pollen development (Wan *et al*., 2020). To rapidly enrich the genetic resources of lipid metabolic GMS genes in maize, we combined anther transcriptome analysis and CRISPR/Cas9 genome editing technology to generate mutants of 14 potential candidate genes (Figures 1 and S1–S3). By phenotypic analysis, we discovered that *ZmTKPR1‐1* and *ZmTKPR1‐2* are new maize GMS genes, as simultaneous mutagenesis of both genes resulted in complete male sterility (Figure 2b). In contrast, the single homozygote mutants of 14 lipid metabolic genes either exhibited no male sterility or only slight male sterility. The fact that the mutant does not show an obvious phenotype is probably due to genetic robustness (El‐Brolosy *et al*., 2019). Among several proposed mechanisms underlying genetic robustness, functional redundancy is considered to be a crucial one (Hu *et al*., 2023; Tautz, 1992). In this study, *ZmTKPR1‐1* and *ZmTKPR1‐2* are paralogous genes, and only their double mutants exhibited complete male sterility, reinforcing the functional redundancy mechanism. Further, no highly similar paralogues were found in the other 12 lipid genes (data not displayed in this paper). Rewiring of genetic networks (Barabási and Oltvai, 2004) and genetic compensation (El‐Brolosy *et al*., 2019; Rossi *et al*., 2015) are two other underlying mechanisms of genetic robustness, which may explain the male fertile phenotype of single mutants of the 12 lipid genes without paralogues. Therefore, integrating anther transcriptome analysis, functional redundancy analysis and gene editing is an effective strategy for discovering new male sterile genes.

### Functional diversification of *TKPR1* genes in anther and pollen development among different plants

*ZmTKPR1‐1* and *ZmTKPR1‐2*, similar to their orthologues *AtTKPR1* and *OsTKPR1*, were found to play conserved roles in controlling male fertility, supporting the functional conservation of lipid metabolic GMS genes among different plants (Gomez *et al*., 2015). Nevertheless, our results highlight the diversified and different functions of *TKPR1* genes in controlling lipid metabolism and male fertility in different species. The single‐gene mutants of *ZmTKPR1‐1* or *ZmTKPR1‐2* are male fertile in maize, whereas single‐gene mutants of *OsTKPR1* (the closest orthologue of *ZmTKPR1‐2*) are completely male sterile in rice (Xu *et al*., 2019), and *Os08g0515900*, the closest orthologue to *ZmTKPR1‐1*, may not be required for male fertility in rice.

In addition, AtTKPR1 and OsTKPR1 are only localized in the ER, while ZmTKPR1‐1/‐2 are localized in both the ER and nuclei (Figures 4c and S6a,b). The nuclear localizations of ZmTKPR1‐1/‐2 suggest that they may act similarly to TFs or regulators. To date, the multiple functions of several enzymes have been identified as transcriptional activators (Ke *et al*., 2020; Wang *et al*., 2023), but nuclear‐localized TKPR has not been found in other plants. OsUGE1, a UDP‐glucose epimerase, can act as a transcriptional activator to promote anther tapetal degradation in rice (Wang *et al*., 2023). Our results also revealed that tapetal PCD was delayed in *tkpr1‐1/‐2* anther (Figure S4c). Whether there is a new regulatory mechanism for *ZmTKPR1‐1/‐2* on maize reproductive development still needs further investigation.

### Multiple approaches identify protein–protein interaction to define a sporopollenin metabolon in plant anther

Metabolons are transient enzyme–enzyme assemblies of sequential enzymes that mediate substrate channelling to enhance metabolic efficiency (Fernie *et al*., 2018; Srere, 1985). A prerequisite to defining such enzyme associations as metabolons is to prove their PPIs (Zhang and Fernie, 2021). Considering that the detection of such transient and dynamic enzyme complexes is more difficult than that of stable protein complexes, multi‐approaches are required to test such PPIs, including biochemical experiments *in vivo* and *in vitro* and structure‐based methods (Norris *et al*., 2022; Xu *et al*., 2023; Yu *et al*., 2023). In *Arabidopsis*, rice and *Brassica napus*, the PPIs in the sporopollenin metabolon consisting of ACOS‐PKS‐TKPR have been identified by Y2H, BiFC, CoIP, GST‐pulldown and FLIM/FRET assays (Lallemand *et al*., 2013). However, structure‐based PPI analysis has not been used in this metabolon. In this study, in addition to Y2H, BiFC and CoIP tests, we confirmed the pairwise interactions between ZmPKSB, ZmTKPR1‐1 and ZmTKPR1‐2 based on AlphaFold2 protein structure predictions, molecular docking and site‐directed mutagenesis (Figures 5 and S7). Importantly, we found that seven and four docking sites are indispensable for ZmPKSB‐ZmTKPR1‐2 and ZmTKPR1‐1‐ZmTKPR1‐2 interactions (Figure S7b,c), respectively. Different from that in maize (this study) and rice, BnTKPR1 is not involved in the sporopollenin metabolon in *Brassica napus*, and AtTKPR1 fails to form a homodimer in *Arabidopsis* (Lallemand *et al*., 2013; Qin *et al*., 2016). Therefore, our results reveal both conserved and diversified functions of components in the sporopollenin metabolon between dicots and monocots.

### The ZmPKSB‐ZmTKPR1‐1/‐2 metabolon is required for the biosynthesis of the reduced tetraketide α‐pyrones for pollen exine formation

Owing to its extreme chemical and physical recalcitrance, sporopollenin is the toughest material in the plant kingdom (Montgomery *et al*., 2016). For decades, the determination of sporopollenin components has become a challenging task. Recent genetic studies and analytical characterization of sporopollenin through solid‐state NMR and targeted degradation techniques have found that polyhydroxylated α‐pyrone subunits and hydroxylated aliphatic units are the curial components of sporopollenin (Grienenberger and Quilichini, 2021; Li *et al*., 2019; Mikhael *et al*., 2020). Our data indicate that ZmTKPR1‐1/‐2 has similar reduction activities to their orthologues, AtTKPR1 and OsTKPR1, and can produce reduced tetraketide α‐pyrones (belong to polyhydroxylated α‐pyrones), suggesting that the loss‐of‐function mutation of *TKPR1‐1/‐2* leads to a lack of this core component of sporopollenin. Thus, *tkpr1‐1/‐2* microspores exhibit very thin and completely disorganized exine without foot layer, baculua and tectum, which is consistent with the functions of ZmTKPR1‐1/‐2 in sporopollenin biosynthesis. Interestingly, while the *pksb* mutation reduces the expression of *ZmTKPR1‐1/‐2*, the pollen exine of *pksb* has a foot layer, baculua and tectum (Figures 5e, 7a,b and S7e). For this possible reason, we speculate that in the *pksb* anther, the reduced expression of *ZmPKSA‐1/‐2* (paralogues of *ZmPKSB*) may still have polyketide synthase activities (Liu *et al*., 2022), which could produce a small amount of polyhydroxylated α‐pyrones. In *Arabidopsis*, the nearly identical defective pollen exine of the *pksa/b* double mutant and the *tkpr1* mutant supports the above inference (Grienenberger *et al*., 2010). Further studies may confirm the hypothesis by creating a *pksa*/*b* double mutant in maize.

### Different effects of *ZmPKSB* and *ZmTKPR1‐1/‐2* on lipid metabolism for anther cuticle formation

The anther cuticle is a crucial lipid layer covering the outer surface of the anther epidermis. Although previous studies have shown that anther cuticle formation and pollen exine formation share certain common lipid biosynthetic pathways (Ariizumi and Toriyama, 2011; Shi *et al*., 2015; Wan *et al*., 2020), there is still a large gap in the mechanism underlying anther cuticle formation compared with pollen exine formation. In particular, different lipid genes involved in the same biosynthesis pathway but with different roles in anther cuticle formation have rarely been systematically compared.

ZmPKSB and ZmTKPR1‐1/‐2 can form a protein complex that increases their sequential catalytic activities (Figures 5 and 6), but their mutants exhibit opposite defective cytological phenotypes of the anther cuticle (Figure 7b). The possible reasons are as follows. First, *ZmPKSB* has a trade‐off function on anther cuticle and pollen exine formation (Liu *et al*., 2022), but not for *ZmTKPR1‐1/‐2*. All nine cutin/wax‐related genes were significantly up‐regulated in *pksb* anthers (Liu *et al*., 2022), but four of these genes (*ZmKCAS‐4*, *ZmGL8*, *ZmCER8* and *ZmABCG2a*) were significantly down‐regulated in *tkpr1‐1/‐2* anthers (Figure 7e). Correspondingly, the total contents of anther cutin and wax in *tkpr1‐1/‐2* anthers were significantly lower than those in *pksb* anthers (Figure 7c,d). Second, since ZmPKSB and ZmTKPR1‐1/‐2 are located upstream and downstream of a sequential enzymatic reaction (Figure 6c,e), respectively, their mutations may lead to the accumulation of different intermediate lipid products. For example, according to the phenylpropanoid pathway for biosynthesis of sporopollenin precursors (Wan *et al*., 2020), loss‐of‐function of *ZmPKSB* probably causes excessive accumulation of midchain‐ and ω‐OH fatty acyl‐CoA substrates (Liu *et al*., 2022). ω‐OH fatty acids (HFAs) have been shown to be the common substrates for the biosynthesis of anther cutin monomers and pollen sporopollenin precursors (Djukanovic *et al*., 2013; Dobritsa *et al*., 2009; Li *et al*., 2010; Yi *et al*., 2010). Thus, excess ω‐OH fatty acyl‐CoAs may be used to generate more ωHFA for anther cuticle formation when pollen exine formation is blocked in *pksb* anthers (Liu *et al*., 2022). The contents of cutin monomers C16 ωHFA and C18: 1 ωHFA in *pksb* anthers were significantly higher than those in *tkpr1‐1* and WT anthers (Figure 7c), further supporting the above speculation. Finally, lipid metabolism in the anthers of flowering plants is a very complex biological process, and its underlying mechanism remains unclear (Wan *et al*., 2020). The lipid products produced by these lipid metabolic GMS genes have a myriad of diverse functions. Instead of simple single lines downward, the catalytic processes of these enzymes are actually intertwined and eventually form pollen walls, anther cuticles and membrane structures of subcellular organelles, which requires further investigation.

## Materials and methods

### Plant materials, growth conditions and phenotype characterization

Maize inbred lines B73, Zheng 58 and M6007 and hybrid Hi II were used in this study. B73 and M6007, corresponding to the WT lines of maize GMS mutants *lob30* and *ms7‐6007*, respectively, were originally obtained from the Maize Genetics Cooperation Stock Center (http://maizecoop.cropsci.uiuc.edu). Zheng58 and Hi II are maintained in our laboratory. All plants were grown in the experimental stations of the University of Sciences and Technology Beijing (USTB) in Beijing and Sanya, excepting T_0_ transgenic plants that were grown in a greenhouse under long‐day conditions (16 h/8 h (day/night) at 26 °C/22 °C). A Canon EOS 700D digital camera and an SZX2‐ILLB stereomicroscope (Olympus, Japan) were used to photograph the images of tassels and anthers, respectively. Pollen grains were stained with a 1% I_2_‐KI solution and captured using an Olympus SZ51 microscope (Olympus, Japan) (Zhang *et al*., 2018a).

### RNA‐seq data analysis

Anthers were collected from B73, Zheng58 and M6007 maize lines at 11 stages (stages 5, 6, 7, 8a, 8b, 8b–9, 9, 9–10, 10, 11 and 12). The classifications of developmental stages of the collected anthers were carried out as described previously (Wan *et al*., 2019). The total RNA was purified using poly‐T oligo‐attached magnetic beads, and paired‐end sequencing (2 × 150 bp) was performed on the Illumina Hiseq 4000 platform. Using TopHat 2.0 with default parameters (Trapnell *et al*., 2009), the clean reads were mapped to the B73 reference genome (AGPv4). Gene expression levels were calculated and normalized into RPKM (reads per kb per million mapped reads) values based on annotated maize gene models (Ensembl_release‐37) by Rsubread and edgeR (Liao *et al*., 2014; Robinson *et al*., 2010). Expression levels of the investigated genes are listed in Table S1.

### Plasmid construction, maize transformation and genotyping

For the mutagenesis of 14 maize lipid metabolic genes, the CRISPR/Cas9 plasmids were constructed using the *pBUE411* vector as a backbone (Xing *et al*., 2014). The CRISPR‐P 2.0 (http://crispr.hzau.edu.cn/CRISPR2/) was used to choose specific gRNAs targeting the coding sequences of 14 maize lipid metabolic genes, and off‐target analysis of gRNAs was carried out on the website (http://www.rgenome.net/cas‐offinder/). A CRISPR/Cas9 plasmid contains two gRNAs. For assembly of the two gRNAs, the PCR fragment was amplified based on *pCBC‐MT1T2* with the primer pair specific for each gene (Table S3), and the purified PCR fragment was cloned into a *Bsa*I‐digested *pBUE411* vector.

The CRISPR/Cas9 recombinant plasmids were used for *Agrobacterium tumefaciens*‐mediated transformation into maize (Hi‐II) following the previously published protocols (Frame *et al*., 2002). The positive transformants were selected by PCR amplification using primers Bar‐F and Bar‐R. The genotyping of transgenic progenies in T_0_, F_1_ and F_2_ generations was performed as described previously (Jiang *et al*., 2021a). All primers used in this study are listed in Table S3.

### Cytological analysis and microscopy

For transverse section, SEM and TEM analyses, the fresh anthers of WT and *tkpr1‐1/‐2* from stages S5 to S13 were immersed in FAA solution (Coolabor, China) or 3.5% glutaraldehyde solution overnight according to the description of a previous study (An *et al*., 2020), respectively, and the images were photographed using a BX‐53 microscope (Olympus, Japan), a HITACHI S‐3400N scanning electron microscope (Hitachi Japan) and a HITACHI H‐7500 transmission electron microscope (Hitachi Japan), respectively.

### TUNEL assay

The anthers of WT and *tkpr1‐1/‐2* at different stages were collected and prepared to perform paraffin sections. The selected paraffin sections were dewaxed in xylene and dehydrated in a gradient ethanol series. DNA fragmentation in tapetum was detected by TUNEL assay using a TUNEL kit (DeadEndTM Fluorometric TUNEL System, Promega) according to the manufacturer's instructions. Signals from anther samples were photographed under a fluorescence confocal scanner microscope (TCS‐SP8, Leica).

### Phylogenetic and microsynteny analysis

The 29 orthologues of ZmTKPR1‐1 and ZmTKPR1‐2 in 11 plants were obtained from the EnsemblPlants website (https://plants.ensembl.org). The alignment of amino acid sequences was performed with DNAMAN8 software. The phylogenetic tree was constructed using MEGA11 software with the neighbour‐joining method (Tamura *et al*., 2021).

For the microsynteny analysis, we first identified the neighbouring genes of *ZmTKPR1‐1* and *ZmTKPR1‐2* in the B73 maize reference genome (AGPv5). Additionally, we obtained the flanking genes of *ZmTKPR1‐1* and *ZmTKPR1‐2* orthologues in *Arabidopsis*, rice, sorghum and wheat from the Phytozome V12 database (https://phytozome.jgi.doe.gov/pz/portal.html). To determine the microsynteny of *ZmTKPR1‐1* and *ZmTKPR1‐2*, we conducted multiple sequence alignments around the flanking genes of *ZmTKPR1‐1* and *ZmTKPR1‐2*, as well as their orthologues in three monocot species and one eudicot species, using the MCScan software.

### Subcellular localization of ZmTKPR1‐1 and ZmTKPR1‐2

The full‐length coding sequences (CDS) of *ZmTKPR1‐1* and *ZmTKPR1‐2* were amplified by PCR, and the resulting fragments were inserted into the expression vectors *pUC19* and *pJG185* using the infusion method. The recombinant *pUC19* vectors were co‐transformed into maize protoplasts with HDEL‐mCherry (an endoplasmic reticulum (ER) marker) and GHD7‐mCherry (a nuclear marker) (Nelson *et al*., 2007). The recombinant pJG185 vectors were transiently expressed in tobacco leaves with ZmMs30‐YFP, an ER marker (An *et al*., 2019). DAPI staining was used as a nuclear marker. The GFP‐, YFP‐, or mCherry‐tagged fluorescent signals were detected using a fluorescence confocal scanner microscope (TCS‐SP8, Leica).

### Quantitative real‐time PCR (qPCR) analysis

Total RNA was extracted using TRIzol reagent (Invitrogen), and DNase I (Promega) was used to eliminate genomic DNA contamination. Subsequently, cDNA synthesis was performed using 5× All‐In‐One RT MasterMix (ABM, Canada) according to the manufacturer's instructions. Quantitative real‐time PCR (qRT‐PCR) was carried out on a QuantStudio 5 Real‐Time PCR system (ABI) using TB GreenTM Premix EX TagTM (TakaRa, Japan) and the corresponding primer set (Table S3). *ZmCyanase* (*Zm00001d032736*) and *ZmUbi2* (*Zm00001d05383*) were employed as internal control genes. Each sample was replicated three times biologically, with each replicate performed in triplicate technically. The 2^−ΔΔCt^ method was used to analyse the amplification data, and the results are presented as means ± standard deviation (SD).

### Transient dual‐luciferase assay

The cloning of the promoter regions of *ZmTKPR1‐1* and *ZmTKPR1‐2* (2000 bp and 2224 bp upstream of the ATG start codon respectively) into the *pEASY‐LUC* vector using homologous recombination yielded the reporter constructs *proZmTKPR1‐1: LUC* and *proZmTKPR1‐2: LUC*. Additionally, the CDS region of *ZmMYB84* (*Zm00001d025664*) was inserted into the *pRTBD* vector, generating the effector construct *35S*‐Ω: *ZmMYB84*. Maize protoplast transformation was then performed, and the relative LUC activity was measured as previously described (Hou *et al*., 2023).

### Electrophoretic mobility shift assay (EMSA)

The full‐length coding sequence (CDS) of *ZmMYB84* was fused with the carboxyl terminal of maltose‐binding protein (MBP) and cloned into the vector *pMCSG7*. Biotin‐labelled *ZmTKPR1‐1* and *ZmTKPR1‐2* promoter probes were generated by annealing primer pairs ZmTKPR1‐1bio‐F/ZmTKPR1‐1bio‐R and ZmTKPR1‐2bio‐F/ZmTKPR1‐2bio‐R, respectively. The fusion vector transformation, protein purification and EMSA were carried out following the methods described in a previous study (Hou *et al*., 2023). Excess core motif‐mutated probes were used as competitors to test the specificity of the DNA–protein interaction.

### Structure prediction

The amino acid sequences of ZmPKSB, ZmTKPR1‐1 and ZmTKPR1‐2 were used to generate FASTA files, which served as input for structure prediction using Alphafold 2.1.1 (Jumper *et al*., 2021). The resulting PDB files were visualized using Pymol (Alexander *et al*., 2011). Pymol was also employed for structural alignments of ZmPKSB, ZmTKPR1‐1 and ZmTKPR1‐2's beta barrel structures with the PH‐domain of FERM1 from PDB entry 1MIX. Additionally, the AlphaFold Protein Structure Database (Jumper *et al*., 2021; Varadi *et al*., 2022) offers a similar structure prediction for ZmPKSB, ZmTKPR1‐1 and ZmTKPR1‐2.

### Y2H assay

The matchmaker GAL4 Two‐Hybrid System (Clontech) was employed to investigate PPIs. The full‐length coding sequences of *ZmPKSB*, *ZmTKPR1‐1* and *ZmTKPR1‐2* were cloned into *pGADT7* and *pGBKT7* vectors. Amino acid substitution vectors, including *pGBKT7‐ZmTKPR1‐1‐9M*, *pGBKT7‐ZmTKPR1‐2‐7M* and *pGADT7‐ZmTKPR1‐1‐4M*, were constructed using fusion PCR. Co‐transformation of a *pGADT7/prey* plasmid and a *pGBKT7/bait* plasmid was performed in yeast strain AH109 and verified on selective media according to the manufacturer's instructions. Negative control vectors, *pGADT7‐T7* and *pGBKT7‐lam*, were used, as well as a positive control with *pGADT7‐T7* and *pGBKT7‐53*. Subsequently, vector co‐transformation into yeast strains and yeast screening were conducted following the methods described in a previous study (An *et al*., 2020).

### BiFC assay

To investigate PPIs, we employed the bimolecular fluorescence complementation (BiFC) assay, following the methods described in a previous study (Walter *et al*., 2004). The full‐length coding sequences (CDSs) of *ZmPKSB*, *ZmTKPR1‐1* and *ZmTKPR1‐2* were fused with the N‐terminal (1–155 amino acids) and C‐terminal (156–239 amino acids) fragments of yellow fluorescent protein (YFP). These fusion constructs were then cloned into the *pUC19‐35S* vector using homologous recombination (Li *et al*., 2005). HDEL‐mCherry, an ER marker, was used in conjunction with the paired recombinant BiFC plasmids. The maize protoplasts were co‐transformed with the BiFC plasmids and the ER marker and incubated in the dark at 28 °C for 12–16 h. YFP fluorescence was subsequently visualized using a confocal laser‐scanning microscope (Leica TCS SP8) with excitation at 514 nm and emission detection at 525–565 nm.

### Co‐IP assay

Co‐immunoprecipitation (Co‐IP) assays were conducted in a protoplast transient expression system. For this purpose, vectors containing *35S‐ZmPKSB‐nYFP‐Flag*, *35S‐ZmTKPR1‐1‐nYFP‐Flag*, *35S‐ZmTKPR1‐2‐nYFP‐Flag*, *35S‐ZmPKSB‐Myc*, *35S‐ZmTKPR1‐1‐Myc* and *35S‐ZmTKPR1‐2‐Myc* were constructed. Maize leaf protoplasts were co‐transformed with these vectors, while *35S‐nYFP‐Flag* and *35S‐ZmbHLH51‐nYFP‐Flag* vectors were used as negative controls. The transformed protoplasts were then incubated in the dark at 28 °C for 16 h. Total protein extraction was performed following the previous protocol (Ma *et al*., 2018). The immunoprecipitation of total protein was carried out using anti‐cMyc affinity gel (E6654; Sigma‐Aldrich) according to the manufacturer's instructions. The eluted immunoprecipitates were subjected to immunoblotting using anti‐cMyc (9E10, sc‐40; Santa Cruz, 1:1000) and anti‐FLAG (FLA‐1, M185‐3, MBL, 1:10 000) antibodies. A secondary goat anti‐mouse‐IgG‐HRP antibody was used at a 1:10 000 dilution in PBS. The signals were detected using a SuperSignal West Pico kit (34080; Thermo Scientific).

### Protein purification, enzymatic analysis and LC–MS/MS analysis

The full‐length CDSs of *ZmTKPR1‐1* and its four mutations (−4aa, S128A, Y164A and K168A), as well as *ZmTKPR1‐2* and its four mutations (−1aa, S130A, Y166A and K170A), were cloned into the pMCSG7 vector, respectively. The vector construction and protein purification procedures followed the methods described in a previous study (Zhang *et al*., 2021). For enzyme activity assays, 10 μg recombinant protein of ZmPKSB was firstly incubated with C16/18‐CoA (0.1 mM) in a reaction buffer (10 mM MgCl_2_, 5 mM ATP, 2.5 mM DDT and 0.1 mM malonyl CoA in 60 mM sodium phosphate buffer) for 30 min at 30 °C to generate triketide and tetraketide. Subsequently, 10 μg ZmTKPR1‐1 or 10 μg ZmTKPR1‐2 and 1 mM NADPH were added and then incubated for another 1 h at 30 °C to generate reduced tetraketide. To measure the enzyme activities of variants, ZmPKSB and C16/18‐CoA were incubated in the reaction buffer at 30 °C for 30 min to generate triketides and tetraketides. After that, the products were equally divided into eight parts, and the recombinant proteins of variants were added to analyse their enzyme activities, respectively. All reactions were stopped with the addition of 20 μL 1 M HCl. Each final product was extracted with 800 μL of ethyl acetate, and the extract was then evaporated overnight in a vacuum.

The experiments utilized an Acquity UPLC system (Waters) connected to a Quattro Premier XE triple quadrupole MS system (Waters Micromass). Reaction products were placed into an Acquity UPLC BEH C18 column (2.1 × 100 mm, 1.7 μm) along with a precolumn (2.1 × 5 mm, 1.7 μm), and then separated through an increasing acetonitrile gradient in water with 0.1% formic acid. The flow rate was set at 0.45 mL/min with gradient conditions being 50%–100% acetonitrile for 25 min, sustained at 100% for 2 min, reduced to 50% for 1 min and then the column was balanced at 50% acetonitrile for 4 min. Full‐scan, selected ion recording, daughter scan and multiple reaction monitoring modes were implemented for the analysis (Zhu *et al*., 2017).

### Analysis of anther cutin, wax and internal lipid

Anthers at stage S13 from both the WT and *tkpr1‐1/‐2* were collected and subjected to freeze‐drying. The calculation of the anther surface and the determination of the content and component of anther wax, cutin and internal lipid were performed as described previously (An *et al*., 2020).

## Conflict of interest

The authors declare no conflicts of interest.

## Author contributions

X.W. and X.A. designed research and supervised the project; Z.L. and J.W. performed RNA‐Seq and data analysis; S.Z., Y.J. and X.L. performed plasmid construction, genetic transformation, CRISPR/Cas9 assay, qPCR analysis and lipidomics analysis; S.Z. and C.F. carried out phenotypic and cytological observations and the TUNEL assay; L.Z. and C.F. performed Y2H, BiFC and Co‐IP; L.Z. performed TDLR and EMSA assays; J.Z. performed structural predication; X.A., Y.J., S.Z., Q.H. and X.W. wrote and revised the paper; X.W. administered the project.

## Supporting information


**Figure S1** The physical maps and target‐site information of CRISPR/Cas9 constructs for editing14 lipid metabolic genes.
**Figure S2** CRISPR/Cas9 mutagenesis and characterization of the derived six lipid metabolic gene mutants and the proportions of aborted pollen grains in the single‐gene mutants of *ZmTKPR1‐1* and *ZmTKPR1‐2*.
**Figure S3** CRISPR/Cas9 mutagenesis and characterization of the derived six metabolic gene mutants.
**Figure S4** Cytological observation and TUNEL assay of WT and *tkpr1‐1/‐2* anthers.
**Figure S5** The amino acid sequence alignment of ZmTKPR1‐1, ZmTKPR1‐2 and their orthologues from 11 plant species.
**Figure S6** Subcellular localization of ZmTKPR1‐1 and ZmTKPR1‐2 in tobacco leaves and qPCR analysis of *ZmTKPR1‐1*, *ZmTKPR1‐2* and *ZmMYB84*.
**Figure S7** Predicted three‐dimensional structures of protein complexes for ZmPKSB, ZmTKPR1‐1 and ZmTKPR1‐2 using AlphaFold2.
**Figure S8** Analysis of anther cutin, wax and internal lipid contents in WT and *tkpr1‐1/‐2* anthers at stage 13.
**Figure S9** Expression of cutin‐ and wax‐related genes in WT and *tkpr1‐1/‐2* anthers.
**Table S1** Transcriptional levels of 14 investigated genes during anther development based on RNA‐seq analysis in three maize lines.
**Table S2** The detailed cutin, wax and internal lipid compositions in WT, *pksb* and *tkpr1‐1/‐2* anthers.
**Table S3** Primers used in this study.
