# Supplementary material for: CRISPR/Cas9‐based genome editing of 14 lipid metabolic genes reveals a sporopollenin metabolon ZmPKSB‐ZmTKPR1‐1/‐2 required for pollen exine formation in maize
Source: Plant Biotechnol J. 2023 Oct 4;22(1):216–32. doi: 10.1111/pbi.14181 (PMC10754010; doi:10.1111/pbi.14181)
Supplement: Supplementary file 1 — Figure S1 The physical maps and target‐site information of CRISPR/Cas9 constructs for editing14 lipid metabolic genes. Figure S2 CRISPR/Cas9 mutagenesis and characterization of the derived six lipid metabolic gene mutants and the proportions of aborted pollen grains in the single‐gene mutants of ZmTKPR1‐1 and ZmTKPR1‐2. Figure S3 CRISPR/Cas9 mutagenesis and characterization of the derived six metabolic gene mutants. Figure S4 Cytological observation and TUNEL assay of WT and tkpr1‐1/‐2 anthers. Figure S5 The amino acid sequence alignment of ZmTKPR1‐1, ZmTKPR1‐2 and their orthologues from 11 plant species. Figure S6 Subcellular localization of ZmTKPR1‐1 and ZmTKPR1‐2 in tobacco leaves and qPCR analysis of ZmTKPR1‐1, ZmTKPR1‐2 and ZmMYB84. Figure S7 Predicted three‐dimensional structures of protein complexes for ZmPKSB, ZmTKPR1‐1 and ZmTKPR1‐2 using AlphaFold2. Figure S8 Analysis of anther cutin, wax and internal lipid contents in WT and tkpr1‐1/‐2 anthers at stage 13. Figure S9 Expression of cutin‐ and wax‐related genes in WT and tkpr1‐1/‐2 anthers. Table S1 Transcriptional levels of 14 investigated genes during anther development based on RNA‐seq analysis in three maize lines. Table S2 The detailed cutin, wax and internal lipid compositions in WT, pksb and tkpr1‐1/‐2 anthers. Table S3 Primers used in this study. [file PBI-22-216-s001.docx]

**Nine Supplementary Figures and Three Supplementary Tables**

**CRISPR/Cas9-based genome editing of 14 lipid metabolic genes reveals a sporopollenin metabolon ZmPKSB-ZmTKPR1-1/-2 required for pollen exine formation in maize**

Xueli An^1,2,3,4†^, Shaowei Zhang^1,^^3†^, Yilin Jiang^1,3†^, Xinze Liu^1,3^, Chaowei Fang^1,3^, Jing Wang^1,3^, Lina Zhao^1,3^, Quancan Hou^1,3^, Juan Zhang^1,2,3^, Xiangyuan Wan^1,2,3,4^*

^1^ Research Institute of Biology and Agriculture, University of Science and Technology Beijing, Beijing 100083, China.

^2^ Industry Research Institute of Biotechnology Breeding, Yili Normal University, Yining 835000, China.

^3^ Zhongzhi International Institute of Agricultural Biosciences, Beijing 100083, China.

^4^ Beijing Engineering Laboratory of Main Crop Bio-Tech Breeding, Beijing International Science and Technology Cooperation Base of Bio-Tech Breeding, Beijing Solidwill Sci-Tech Co. Ltd., Beijing 100192, China.

^†^ Xueli An, Shaowei Zhang, and Yilin Jiang contribute equally to this work.

* Correspondence: Xiangyuan Wan ([wanxiangyuan@ustb.edu.cn](mailto:wanxiangyuan@ustb.edu.cn))

Prof. Xiangyuan Wan:

Address: Research Institute of Biology and Agriculture, University of Science and Technology Beijing, Beijing 100083, China.

Tel: 86-186-0056-1850; Fax: 86-10-82346928

**
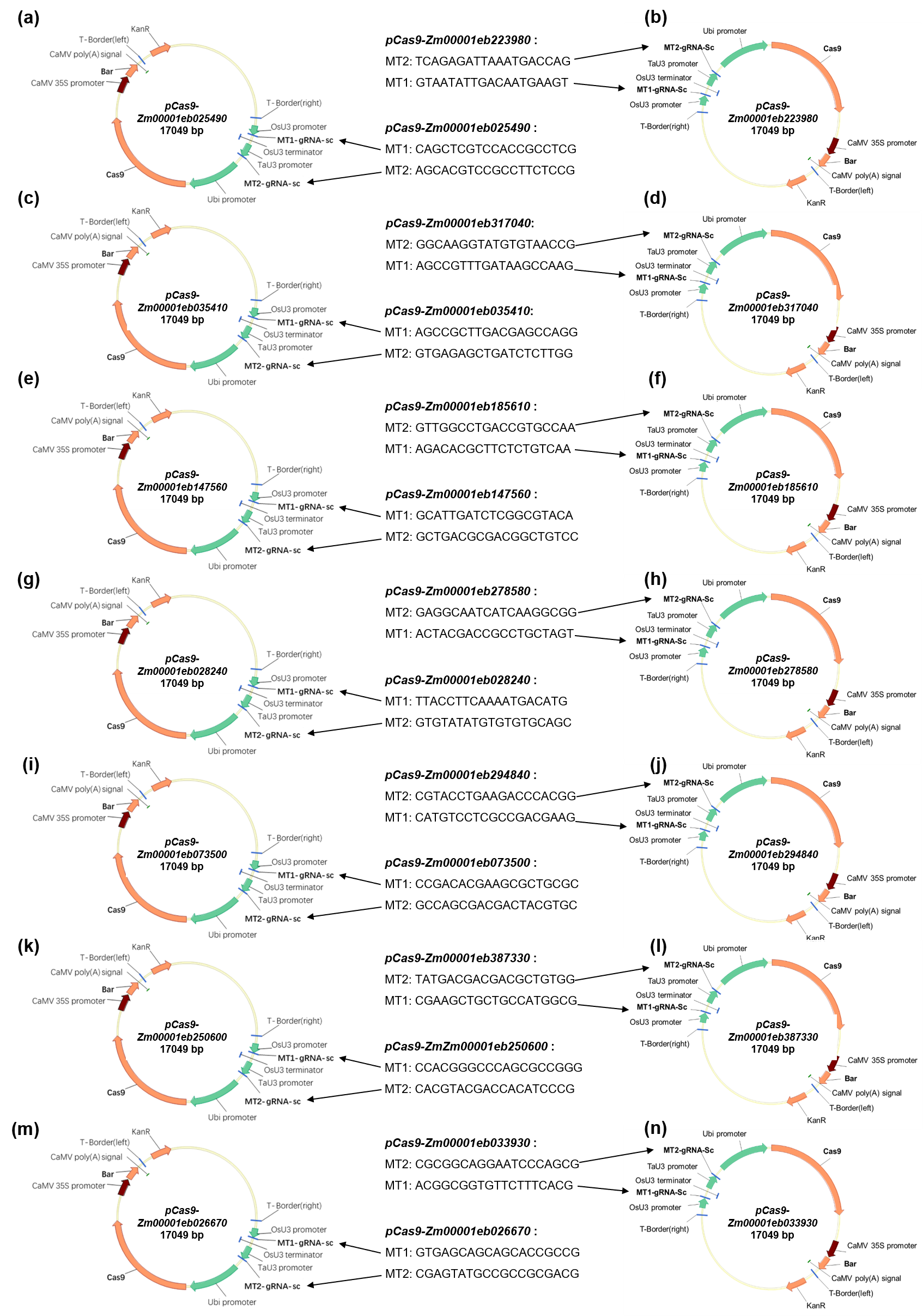
**

**Figure S1** The physical maps and target-site information of CRISPR/Cas9 constructs for editing 14 lipid metabolic genes.

**(a)** Physical map of *pCas9-Zm00001eb025490* construct carrying two gRNAs and the information of target sites in *Zm00001eb025490.*

**(b)** Physical map of *pCas9-Zm00001eb223980* construct carrying two gRNAs and the information of target sites in *Zm00001eb223980.*

**(c)** Physical map of *pCas9-Zm00001eb035410* construct carrying two gRNAs and the information of target sites in *Zm00001eb035410.*

**(d)** Physical map of *pCas9-Zm00001eb317040* construct carrying two gRNAs and the information of target sites in *Zm00001eb317040.*

**(e)** Physical map of *pCas9-Zm00001eb147560* construct carrying two gRNAs and the information of target sites in *Zm00001eb147560.*

**(f)** Physical map of *pCas9-Zm00001eb185610* construct carrying two gRNAs and the information of target sites in *Zm00001eb185610.*

**(g)** Physical map of *pCas9-Zm00001eb028240* construct carrying two gRNAs and the information of target sites in *Zm00001eb028240.*

**(h)** Physical map of *pCas9-Zm00001eb278580* construct carrying two gRNAs and the information of target sites in *Zm00001eb278580.*

**(i)** Physical map of *pCas9-Zm00001eb073500* construct carrying two gRNAs and the information of target sites in Z*m00001eb073500.*

**(j)** Physical map of *pCas9-Zm00001eb294840* construct carrying two gRNAs and the information of target sites in *Zm00001eb294840.*

**(k)** Physical map of *pCas9-Zm00001eb250600* construct carrying two gRNAs and the information of target sites in *Zm00001eb250600.*

**(l)** Physical map of *pCas9-Zm00001eb387330* construct carrying two gRNAs and the information of target sites in *Zm00001eb387330.*

**(m)** Physical map of *pCas9-Zm00001eb026670* construct carrying two gRNAs and the information of target sites in *Zm00001eb026670.*

**(n)** Physical map of *pCas9-Zm00001eb033930* construct carrying two gRNAs and the information of target sites in *Zm00001eb033930.*


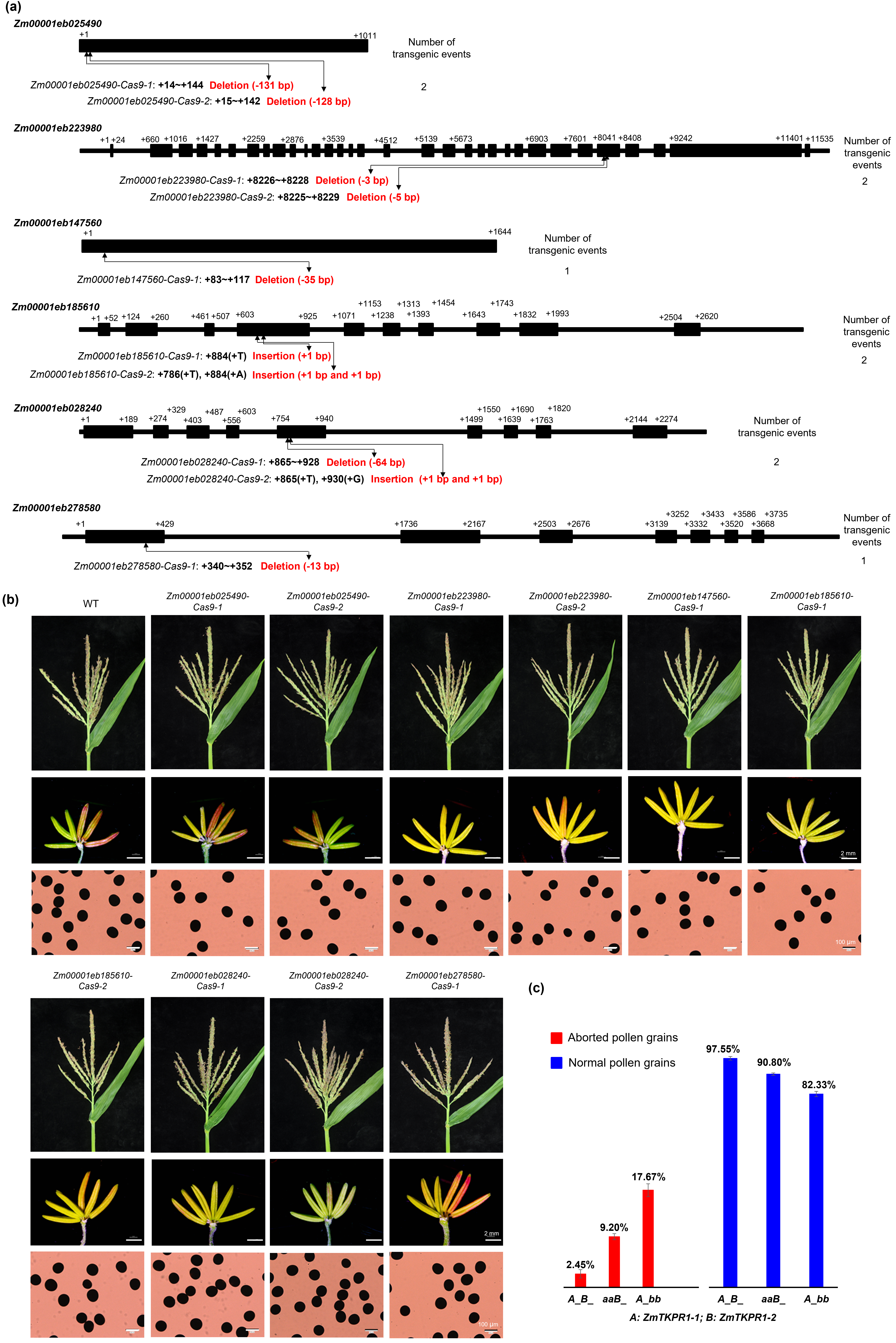


**Figure S2** CRISPR/Cas9 mutagenesis and characterization of the derived six lipid metabolic gene mutants and the proportions of aborted pollen grains in the single-gene mutants of *ZmTKPR1-1* and *ZmTKPR1-2*.

**(a)** Gene structures and mutation analysis of six lipid metabolic genes in WT and the knockout lines generated by the CRISPR/Cas9 genome editing.

**(b)** Phenotypic analyses of tassels, anthers and pollen grains stained with 1% I_2_-KI solution in WT and the knockout lines.

**(c)** The proportions of normal and aborted pollen grains measured by staining with 1% I_2_-KI solution in WT and the single-gene mutants of *ZmTKPR1-1* and *ZmTKPR1-2* at stage13 (n = 3670 to 3920).


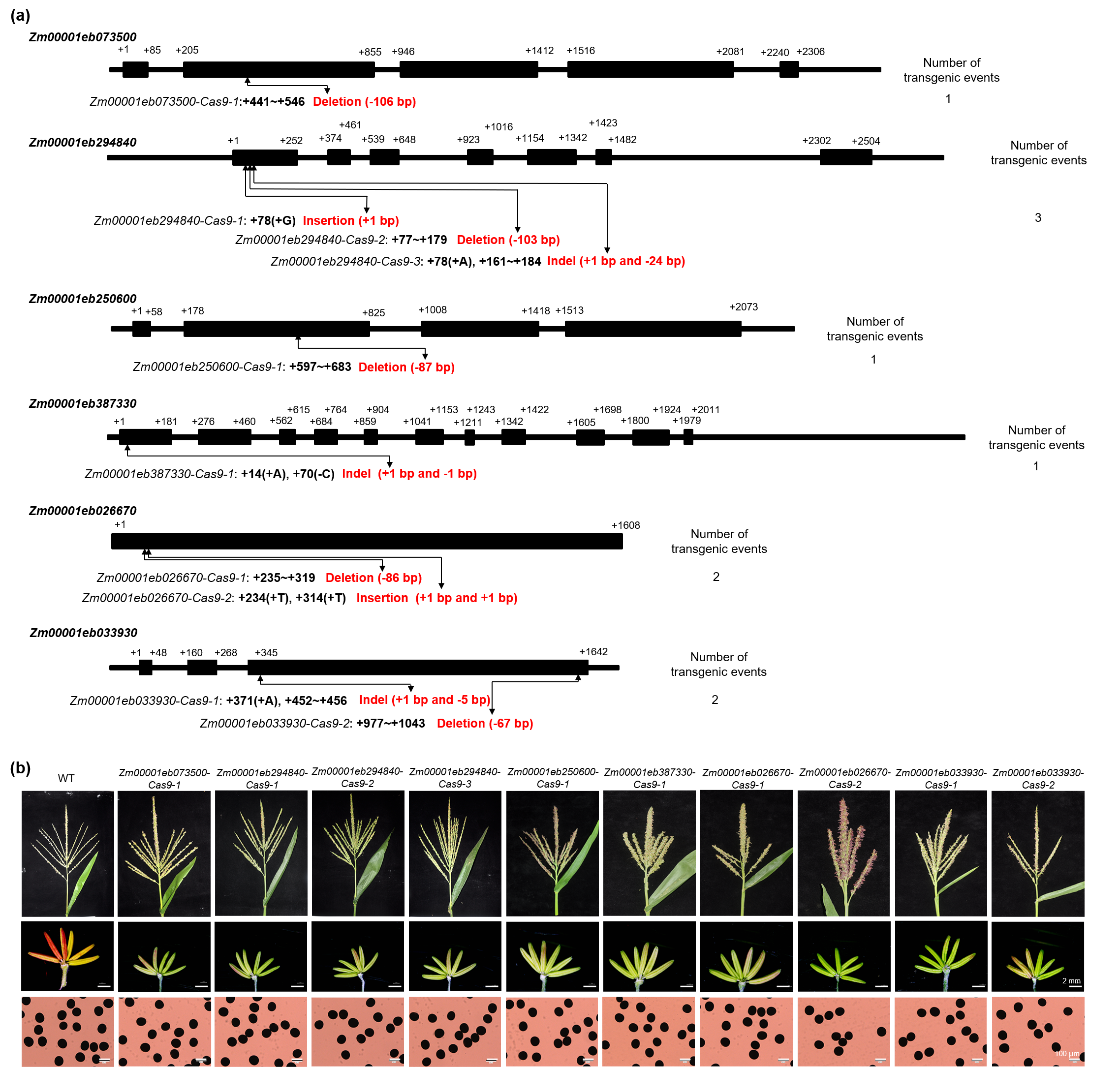


**Figure S3** CRISPR/Cas9 mutagenesis and characterization of the derived six lipid metabolic gene mutants.

**(a)** Gene structures and mutation analysis of six lipid metabolic genes in WT and the knockout lines generated by the CRISPR/Cas9 genome editing.

**(b)** Phenotypic analyses of tassels, anthers and pollen grains stained with 1% I_2_-KI solution in WT and the knockout lines.


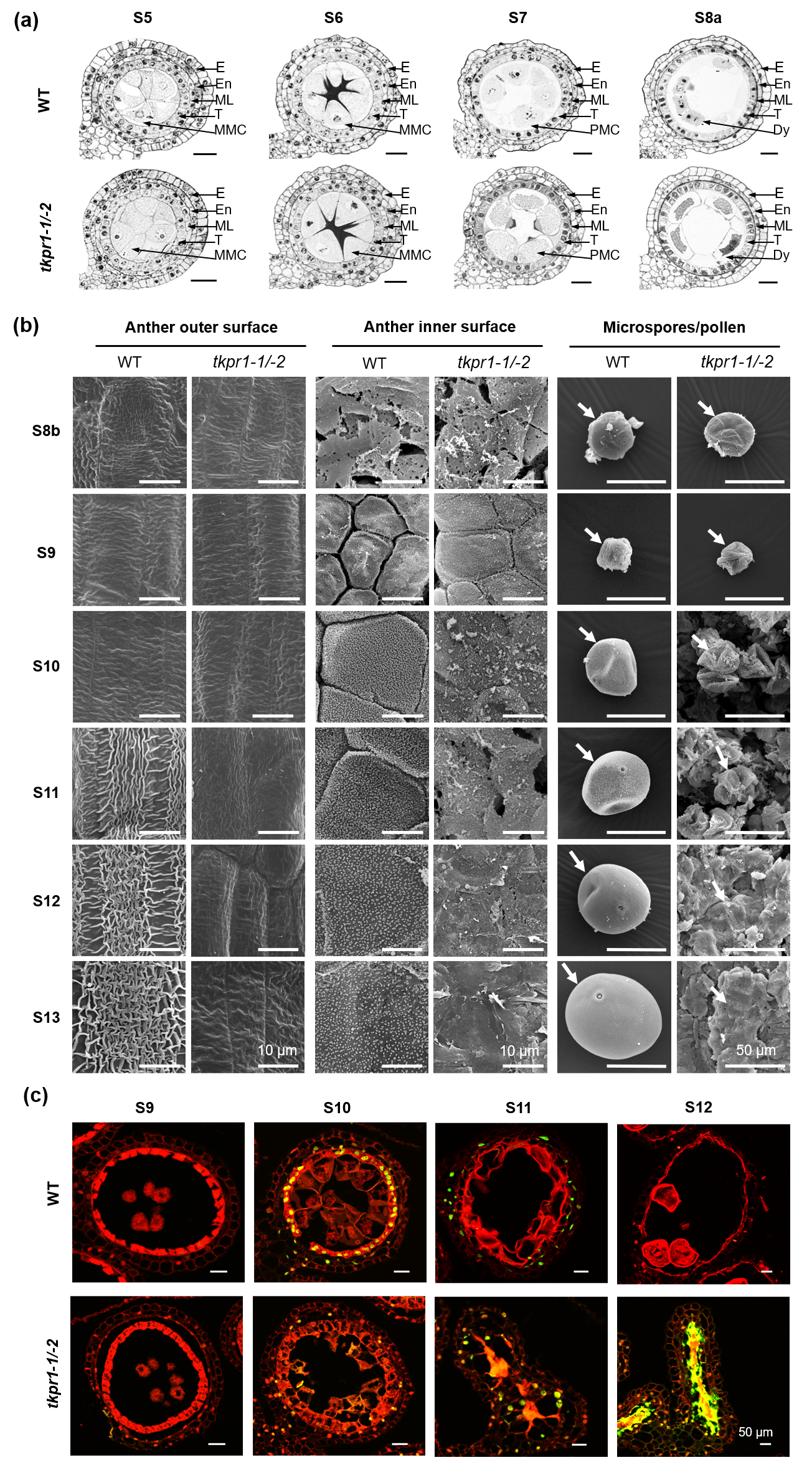


**Figure S4** Cytological observation and TUNEL assay of WT and *tkpr1-1/-2* mutant anthers.

**(a)** Transverse sections of WT and *tkpr1-1/-2* anthers from stages S5 to S8a. E, epidermis; En, endothecium; ML, middle layer; T, tapetum; MMC, Microspore mother cell; PMC, pollen mother cell; Dy, dyad cell.

**(b)** SEM analysis of anther outer surface, inner surface and microspores/pollen in WT and *tkpr1-1/-2* mutant from stages S8b to S13. Microspores/pollen are indicated with white arrows.

**(c)** Detection of DNA fragmentation in tapetal cells by TUNEL assay in WT and *tkpr1-1/-2* anthers from stages S9 to S12.


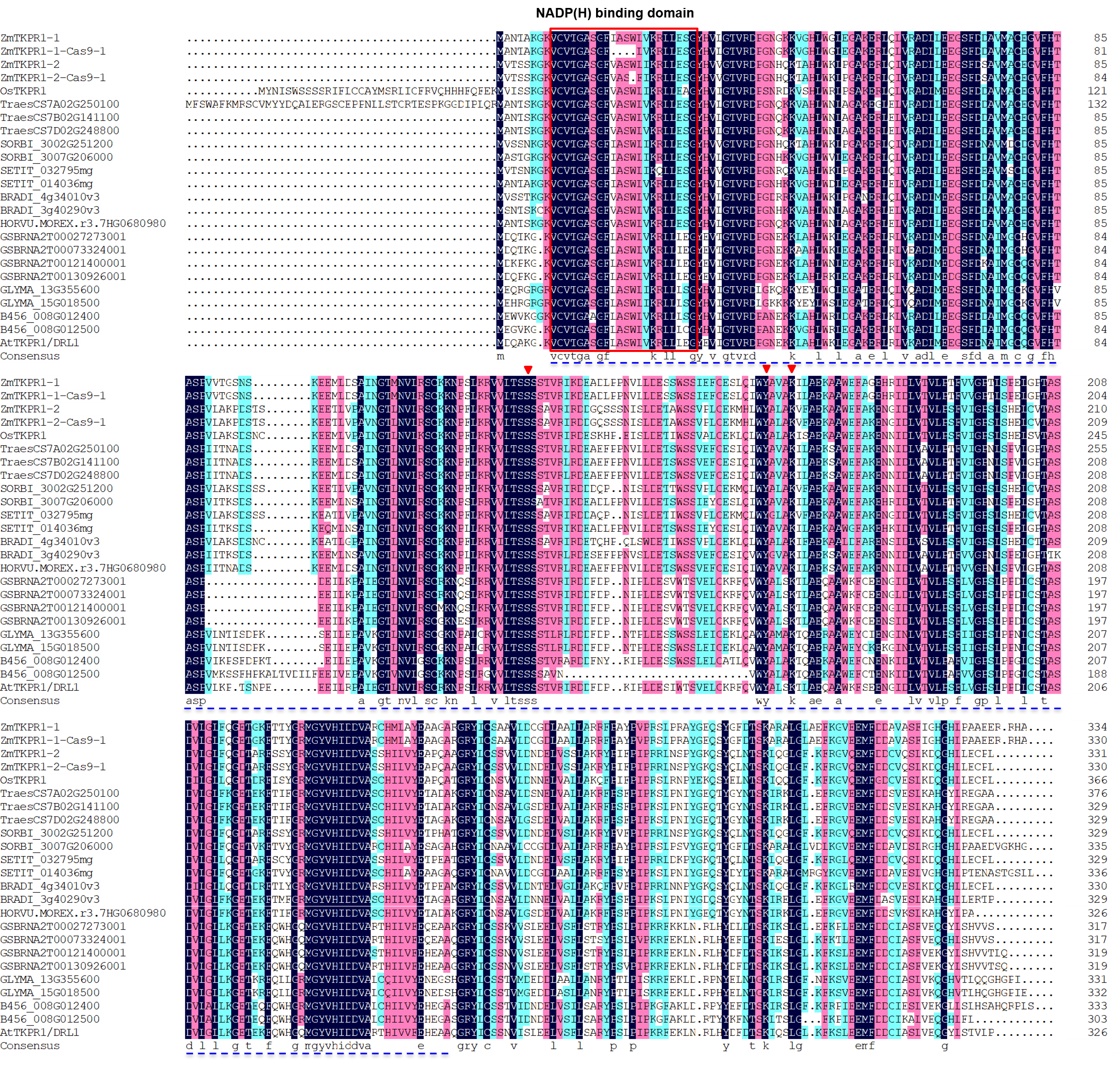


**Figure S5** The amino acid sequence alignment of ZmTKPR1-1, ZmTKPR1-2, and their orthologs from 11 plant species.

The putative common NAD(P)H binding domain is boxed in red. The NAD-dependent epimerase/dehydratase domain is underlined in blue. Predicted catalytic sites are indicated with red arrowheads.


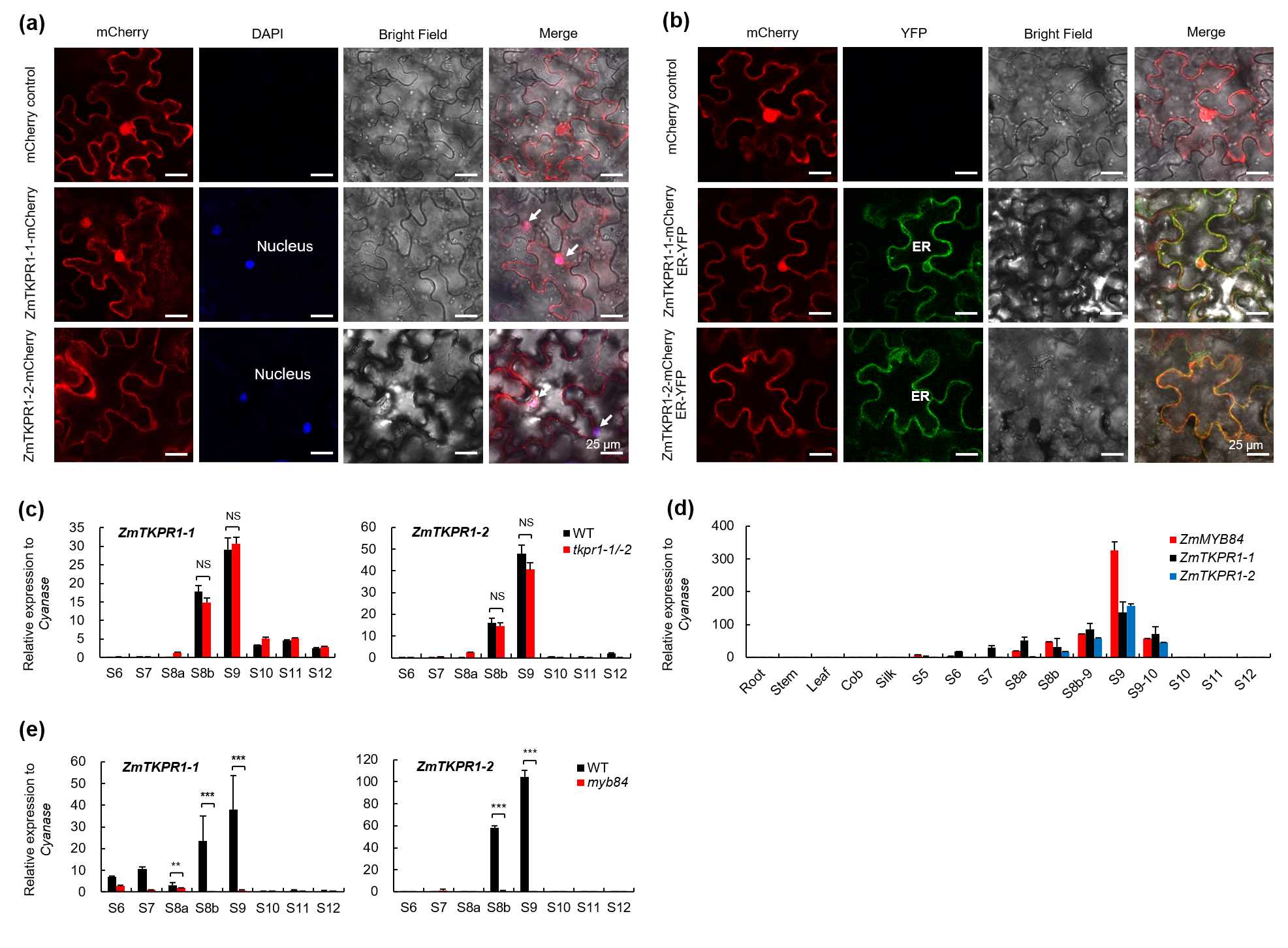


**Figure S6** Subcellular localization of ZmTKPR1-1 and ZmTKPR1-2 in tobacco leaves and qPCR analysis of *ZmTKPR1-1*, *ZmTKPR1-2*, and *ZmMYB84*.

**(a)** Subcellular localization of ZmTKPR1-1 and ZmTKPR1-2 in tobacco leaves. The ZmTKPR1-1-mCherry and ZmTKPR1-2-mCherry vectors were transformed in tobacco leaves, respectively. DAPI staining was used as a nuclear marker. The 35S-mCherry vector was used as a negative control. The nuclei are indicated with white arrows.

**(b)** Subcellular localization of ZmTKPR1-1 and ZmTKPR1-2 in tobacco leaves. The ZmTKPR1-1-mCherry and ZmTKPR1-2-mCherry vectors were co-transformed with the ER-YFP as ER marker, respectively. The 35S-mCherry vector was used as a negative control.

**(c)** qPCR analysis of *ZmTKPR1-1* and *ZmTKPR1-2* in WT and *tkpr1-1/-2* anthers.

**(d)** Spatiotemporal expression of *ZmTKPR1-1*, *ZmTKPR1-2*, and *ZmMYB84* by qPCR analysis.

**(e)** qPCR analysis of *ZmTKPR1-1* and *ZmTKPR1-2* in WT and *myb84* anthers.

NS, **, and *** indicate the significant levels of *P* >0.05, 0.01, and 0.001 determined by a two-tailed Student’s *t*-test, respectively.


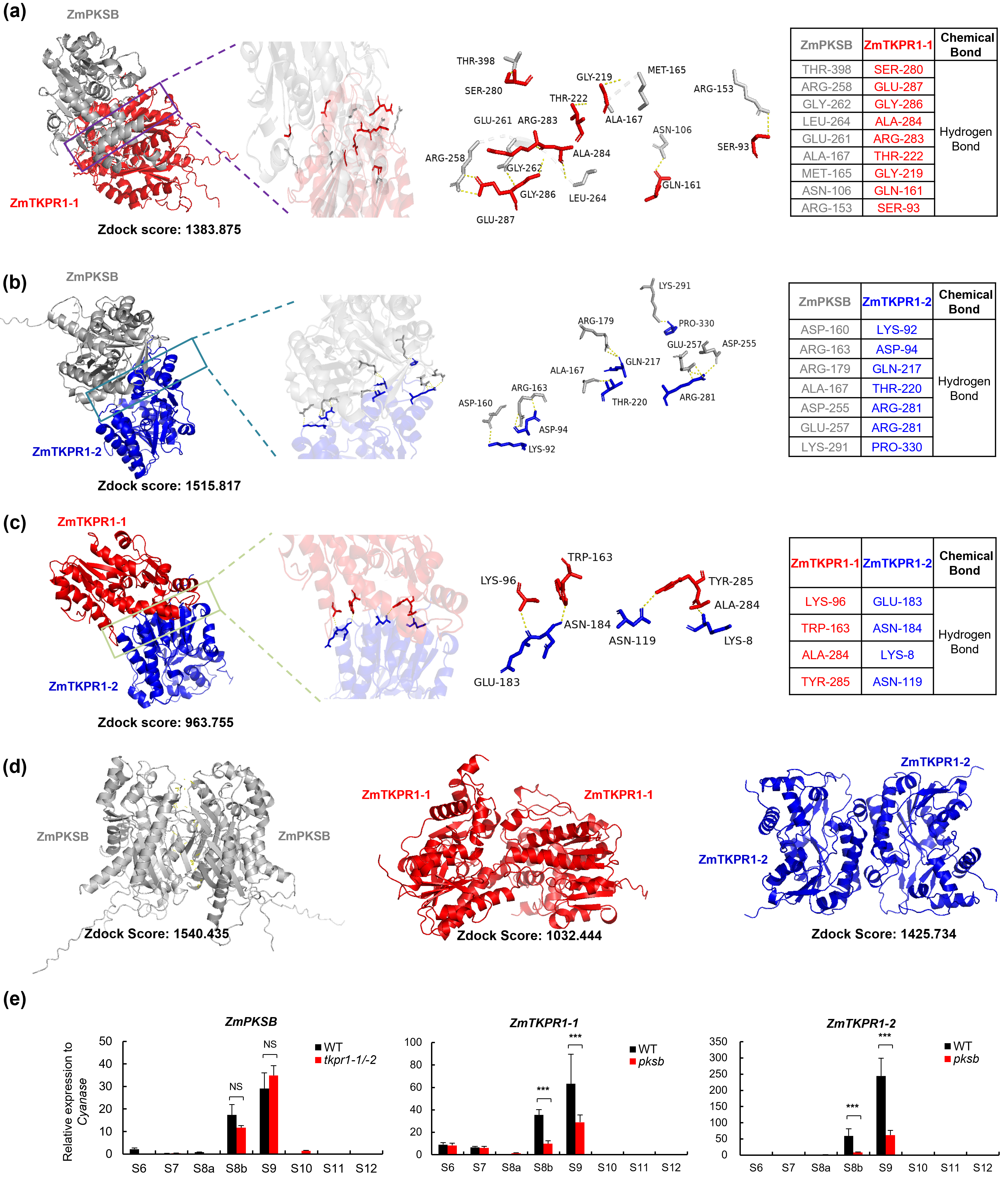


**Figure S7** Predicted three-dimensional structures of protein complexes for ZmPKSB, ZmTKPR1-1, and ZmTKPR1-2 using AlphaFold2.

**(a)** Three-dimensional structure analysis of protein interaction between ZmPKSB and ZmTKPR1-1 and amino acids at the interface forming hydrogen bonds.

**(b)** Three-dimensional structure analysis of protein interaction between ZmPKSB and ZmTKPR1-2 and the screened amino acids forming hydrogen bonds.

**(c)** Three-dimensional structure analysis of protein interaction between ZmTKPR1-1 and ZmTKPR1-2 and the screened amino acids forming hydrogen bonds.

**(d)** Protein-protein interactions predicted of ZmPKSB, ZmTKPR1-1, and ZmTKPR1-2.

**(e)** qPCR analyses of *ZmPKSB* in WT and *tkpr1-1/-2* anthers and of *ZmTKPR1-1* and *ZmTKPR1-2* in WT and *pksb* anthers. NS and *** indicate the significant levels of *P* >0.05 and *P* < 0.001 determined by a two-tailed Student’s *t*-test, respectively.


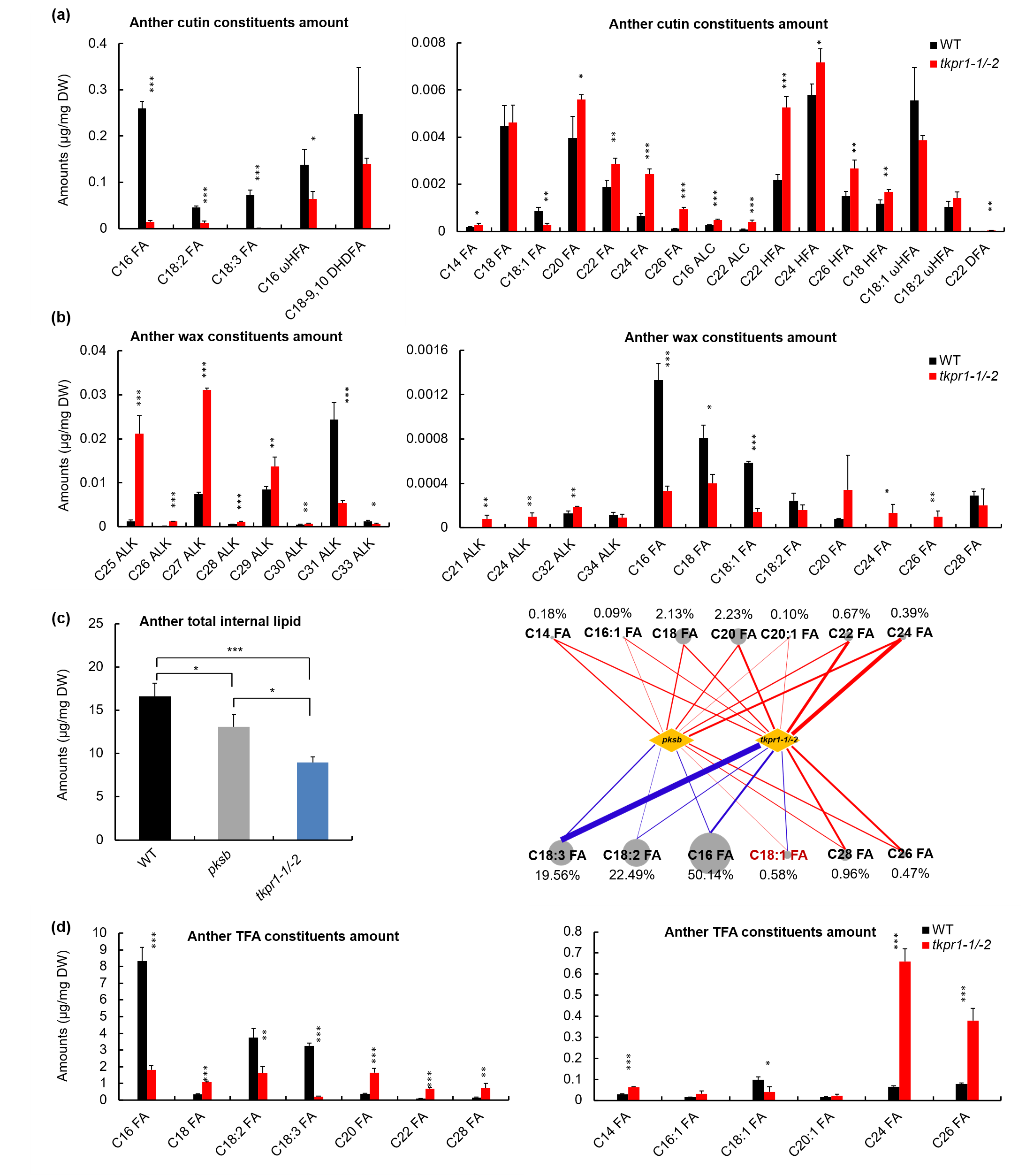


**Figure S8** Analysis of anther cutin, wax and internal lipid contents in WT and *tkpr1-1/-2* anthers at stage S13.

**(a)** The amounts of cutin monomers per unit surface area in WT and *tkpr1-1/-2*.

**(b)** The amounts of wax monomers per unit surface area in WT and *tkpr1-1/-2*.

**(c)** The amounts of internal lipid in WT, *pksb*, and *tkpr1-1/-2* at stage S13. The red or blue lines represent the increase or decrease of the internal lipid in mutant anthers, respectively. The thickness of the lines represents the change magnitude of the increase or decrease.

**(d)** The amounts of internal lipid constituents per unit surface area in WT and *tkpr-1/-2*.

*, **, and *** indicate *P* < 0.05, 0.01, and 0.001 determined by a two-tailed Student’s *t*-test, respectively.


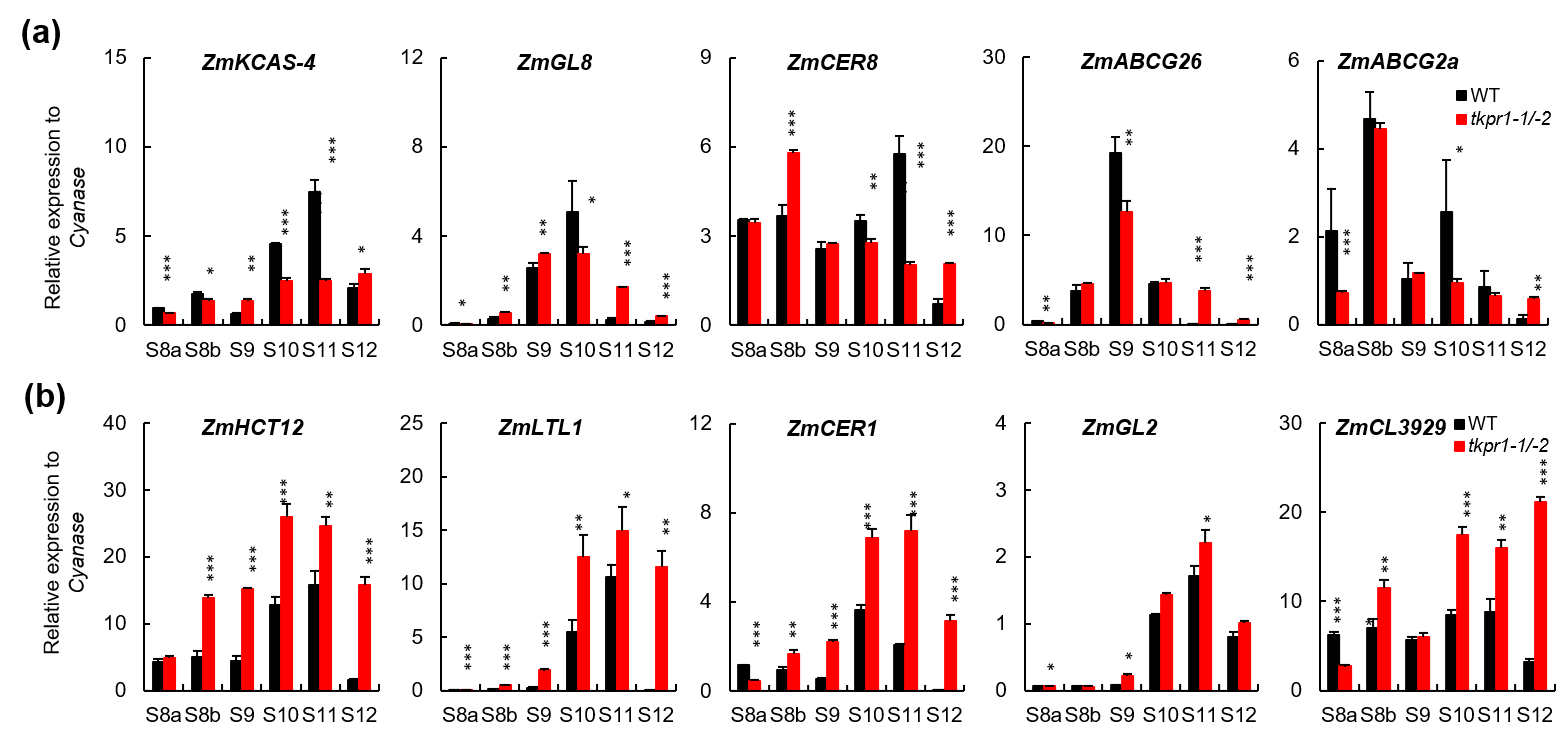


**Figure S9** Expression of cutin- and wax-related genes in WT and *tkpr1-1/-2* anthers.

**(a)** qPCR analysis of five downregulated genes in *tkpr1-1/-2* anthers from stages S8a to S12.

**(b)** qPCR analysis of five upregulated genes in *tkpr1-1/-2* anthers from stages S8a to S12.

*, **, and *** indicate the significant levels of *P* < 0.05, 0.01 and 0.001 determined by a two-tailed Student’s *t*-test, respectively.

**Three Supplementary Tables**

**Table S1:** Transcriptional levels of 14 investigated genes during anther development based on RNA-seq analysis in three maize lines

| **1. Transcriptional levels (RPKM values) of fourteen genes in anther expression data of B73 genetic background** | | | | | | | | | | | | | | |
| --- | --- | --- | --- | --- | --- | --- | --- | --- | --- | --- | --- | --- | --- | --- |
| **Stages** | **Zm00001eb025490** | **Zm00001eb223980** | **Zm00001eb035410** | **Zm00001eb317040** | **Zm00001eb147560** | **Zm00001eb185610** | **Zm00001eb028240** | **Zm00001eb278580** | **Zm00001eb073500** | **Zm00001eb294840** | **Zm00001eb250600** | **Zm00001eb387330** | **Zm00001eb026670** | **Zm00001eb033930** |
| **S5_X1** | 138.04 | 49.10 | 27.65 | 0.37 | 0.00 | 16.03 | 0.31 | 24.92 | 52.38 | 0.70 | 0.20 | 2.68 | 0.00 | 29.89 |
| **S5_X2** | 153.73 | 81.14 | 36.16 | 0.50 | 0.00 | 17.86 | 0.24 | 36.64 | 59.89 | 0.62 | 0.02 | 13.43 | 0.00 | 44.14 |
| **S6_X3** | 322.43 | 95.25 | 144.17 | 0.38 | 0.00 | 17.17 | 0.98 | 28.73 | 65.47 | 0.39 | 0.33 | 102.24 | 0.28 | 84.42 |
| **S6_X4** | 405.33 | 79.75 | 389.34 | 0.49 | 0.00 | 18.38 | 1.34 | 24.79 | 94.53 | 0.64 | 1.70 | 55.30 | 0.23 | 83.93 |
| **S6_X5** | 435.65 | 59.51 | 397.26 | 0.41 | 0.11 | 23.02 | 1.73 | 30.57 | 64.23 | 0.76 | 0.31 | 29.42 | 0.49 | 84.85 |
| **S7_X6** | 293.26 | 95.64 | 544.52 | 0.11 | 0.00 | 13.64 | 1.97 | 30.39 | 61.58 | 0.23 | 0.52 | 0.36 | 0.05 | 184.25 |
| **S7_X7** | 343.62 | 85.56 | 677.28 | 0.21 | 0.07 | 12.96 | 1.29 | 31.45 | 41.42 | 0.10 | 1.31 | 8.28 | 0.00 | 71.88 |
| **S7_X8** | 566.88 | 96.76 | 435.86 | 0.05 | 0.00 | 13.48 | 1.68 | 34.59 | 60.93 | 0.18 | 0.44 | 0.24 | 0.35 | 178.97 |
| **S8a_X12** | 244.16 | 117.07 | 303.41 | 0.39 | 0.00 | 11.81 | 1.17 | 35.97 | 69.01 | 0.95 | 0.34 | 0.42 | 0.06 | 228.50 |
| **S8a_X13** | 47.03 | 144.20 | 141.29 | 42.95 | 0.08 | 12.39 | 0.05 | 18.67 | 16.53 | 2.04 | 0.21 | 0.00 | 0.16 | 64.47 |
| **S8a_X14** | 578.47 | 162.50 | 162.61 | 135.40 | 0.00 | 18.11 | 0.02 | 43.34 | 54.89 | 0.15 | 0.26 | 0.00 | 0.45 | 567.59 |
| **S8b_X18** | 430.44 | 260.92 | 902.27 | 965.33 | 0.00 | 26.86 | 0.04 | 30.66 | 69.61 | 0.03 | 0.21 | 0.38 | 0.16 | 563.19 |
| **S8b_X19** | 446.24 | 220.65 | 534.20 | 418.46 | 0.00 | 22.67 | 0.06 | 30.47 | 90.81 | 0.29 | 0.58 | 0.00 | 0.18 | 499.60 |
| **S8b_X21** | 412.40 | 246.06 | 862.77 | 1255.25 | 0.30 | 22.02 | 0.02 | 37.22 | 59.40 | 0.10 | 0.56 | 0.01 | 0.12 | 581.00 |
| **S8b-9_X20** | 534.85 | 237.30 | 1035.30 | 3319.11 | 17.84 | 28.60 | 0.04 | 62.13 | 40.94 | 0.56 | 0.07 | 0.67 | 0.74 | 631.51 |
| **S8b-9_X22** | 547.53 | 292.94 | 1515.17 | 6376.12 | 195.93 | 30.97 | 0.11 | 65.20 | 73.96 | 0.78 | 0.53 | 0.06 | 0.52 | 410.03 |
| **S8b-9_X23** | 59.83 | 300.03 | 1437.44 | 6195.05 | 115.50 | 27.00 | 0.00 | 33.64 | 19.90 | 1.50 | 0.07 | 0.23 | 0.45 | 187.45 |
| **S9_X24** | 459.37 | 184.80 | 1496.89 | 9967.45 | 78.78 | 60.26 | 18.94 | 117.26 | 49.18 | 0.16 | 1.80 | 16.38 | 2.43 | 888.09 |
| **S9_X25** | 499.42 | 280.49 | 1523.81 | 9672.88 | 257.67 | 45.26 | 2.28 | 75.76 | 111.72 | 0.31 | 0.11 | 2.44 | 0.33 | 490.64 |
| **S9_X26** | 344.03 | 166.27 | 1691.35 | 12959.55 | 81.37 | 46.61 | 4.56 | 117.55 | 56.70 | 0.53 | 0.16 | 35.08 | 4.74 | 564.27 |
| **S9-10_X27** | 203.87 | 26.09 | 188.79 | 1301.74 | 19.16 | 51.77 | 5.10 | 107.08 | 1047.08 | 3.19 | 3389.05 | 1289.81 | 12.62 | 1144.96 |
| **S9-10_X28** | 324.79 | 49.30 | 343.15 | 1931.45 | 34.86 | 62.10 | 15.26 | 105.69 | 1092.19 | 2.69 | 2953.98 | 1162.81 | 8.11 | 1661.35 |
| **S9-10_X29** | 262.26 | 188.31 | 1327.60 | 9179.69 | 161.79 | 51.21 | 11.60 | 85.71 | 72.06 | 1.53 | 81.28 | 119.61 | 3.83 | 676.38 |
| **S10_X30** | 8.01 | 18.22 | 3.77 | 0.58 | 0.00 | 9.03 | 0.07 | 40.92 | 3.92 | 1.18 | 12.30 | 233.40 | 3.09 | 5189.58 |
| **S10_X31** | 100.86 | 24.35 | 5.18 | 2.25 | 0.00 | 13.49 | 0.24 | 47.79 | 25.05 | 0.20 | 67.91 | 458.54 | 1.54 | 4879.39 |
| **S10_X32** | 4.52 | 18.37 | 4.20 | 0.95 | 0.00 | 9.64 | 0.15 | 40.54 | 17.81 | 0.59 | 12.09 | 145.66 | 3.50 | 5284.75 |
| **S11_X33** | 2.90 | 15.26 | 2.50 | 0.26 | 0.03 | 8.28 | 0.23 | 21.23 | 0.04 | 0.24 | 0.52 | 0.00 | 9.90 | 1071.51 |
| **S11_X34** | 2.42 | 20.80 | 2.96 | 0.35 | 0.03 | 10.72 | 0.15 | 72.77 | 0.15 | 0.63 | 0.88 | 11.55 | 8.12 | 3096.29 |
| **S11_X35** | 0.92 | 13.10 | 1.93 | 0.32 | 0.09 | 5.97 | 0.41 | 18.64 | 0.15 | 0.26 | 0.33 | 0.00 | 10.91 | 599.93 |
| **S12_X36** | 1.35 | 11.25 | 1.55 | 0.59 | 0.03 | 4.72 | 0.50 | 19.46 | 0.05 | 0.14 | 0.28 | 0.00 | 6.86 | 407.38 |
| **S12_X37** | 1.16 | 13.26 | 1.46 | 0.68 | 0.02 | 6.29 | 0.56 | 19.50 | 0.04 | 0.11 | 0.18 | 0.00 | 16.17 | 786.77 |
| **S12_X38** | 2.66 | 8.78 | 1.33 | 0.53 | 0.10 | 4.45 | 1.01 | 15.04 | 0.00 | 0.12 | 0.05 | 0.00 | 4.35 | 57.25 |
| **2. Transcriptional levels (RPKM values) of fourteen genes in anther expression data of Zheng58 genetic background** | | | | | | | | | | | | | | |
| **Stages** | **Zm00001eb025490** | **Zm00001eb223980** | **Zm00001eb035410** | **Zm00001eb317040** | **Zm00001eb147560** | **Zm00001eb185610** | **Zm00001eb028240** | **Zm00001eb278580** | **Zm00001eb073500** | **Zm00001eb294840** | **Zm00001eb250600** | **Zm00001eb387330** | **Zm00001eb026670** | **Zm00001eb033930** |
| **S5_X74** | 17.17 | 58.09 | 49.68 | 1.35 | 0.00 | 20.38 | 0.36 | 33.49 | 25.62 | 1.47 | 0.05 | 121.65 | 0.00 | 55.82 |
| **S5_X75** | 72.76 | 38.42 | 12.20 | 0.57 | 0.03 | 19.82 | 1.12 | 33.87 | 39.72 | 0.06 | 0.14 | 2.61 | 0.12 | 31.26 |
| **S5_X76** | 154.55 | 115.12 | 163.56 | 0.11 | 0.03 | 24.92 | 0.34 | 33.31 | 44.72 | 0.07 | 0.26 | 101.21 | 0.39 | 77.57 |
| **S6_X77** | 77.78 | 81.06 | 664.86 | 0.76 | 0.00 | 15.47 | 2.67 | 28.76 | 28.79 | 0.14 | 0.56 | 3.18 | 0.09 | 70.16 |
| **S6_X78** | 142.84 | 58.46 | 604.28 | 0.05 | 0.00 | 29.01 | 3.02 | 38.40 | 41.60 | 0.05 | 0.25 | 20.33 | 0.19 | 169.81 |
| **S6_X79** | 85.48 | 56.83 | 641.64 | 0.02 | 0.00 | 17.66 | 1.65 | 24.24 | 31.08 | 0.29 | 0.24 | 1.27 | 0.37 | 86.20 |
| **S7_X80** | 169.48 | 49.94 | 255.17 | 1.08 | 0.00 | 12.68 | 1.40 | 23.15 | 57.35 | 0.44 | 0.18 | 0.08 | 0.27 | 125.26 |
| **S7_X81** | 542.96 | 45.38 | 131.64 | 0.82 | 0.00 | 19.45 | 0.52 | 33.88 | 107.20 | 0.36 | 0.37 | 0.06 | 0.41 | 179.99 |
| **S7_X82** | 286.52 | 50.73 | 341.16 | 0.23 | 0.00 | 16.69 | 1.69 | 30.50 | 59.04 | 0.14 | 0.17 | 0.84 | 0.14 | 145.12 |
| **S8a_X85** | 51.89 | 90.40 | 44.67 | 0.28 | 0.00 | 12.65 | 0.06 | 21.70 | 10.56 | 0.29 | 0.09 | 0.07 | 0.14 | 20.46 |
| **S8a_X86** | 18.81 | 140.86 | 116.99 | 0.04 | 0.00 | 12.69 | 0.80 | 20.85 | 1.79 | 0.68 | 0.10 | 0.32 | 0.06 | 8.77 |
| **S8a_X87** | 178.39 | 147.43 | 417.93 | 0.19 | 0.00 | 15.48 | 0.27 | 35.25 | 46.60 | 0.22 | 0.14 | 0.00 | 0.02 | 186.09 |
| **S8b_X91** | 166.70 | 233.58 | 1400.74 | 3119.12 | 100.33 | 32.00 | 0.00 | 41.86 | 66.81 | 0.12 | 0.21 | 0.03 | 0.26 | 393.82 |
| **S8b_X92** | 82.12 | 132.18 | 1135.40 | 1056.63 | 0.37 | 26.17 | 0.05 | 42.07 | 89.94 | 28.04 | 103.22 | 33.83 | 0.57 | 217.05 |
| **S8b-9_X90** | 15.28 | 122.15 | 1755.43 | 3032.77 | 16.32 | 31.59 | 0.00 | 23.28 | 3.13 | 0.23 | 0.15 | 0.00 | 0.06 | 131.96 |
| **S8b-9_X94** | 160.01 | 306.70 | 2336.92 | 12336.97 | 457.37 | 76.00 | 0.17 | 87.02 | 44.26 | 0.32 | 0.04 | 0.74 | 1.26 | 376.81 |
| **S8b-9_X95** | 207.46 | 363.04 | 2405.96 | 5867.53 | 59.77 | 35.28 | 0.00 | 100.45 | 66.17 | 0.38 | 0.05 | 0.00 | 0.20 | 300.42 |
| **S9_X96** | 115.55 | 157.22 | 2135.21 | 12867.90 | 124.23 | 57.11 | 0.27 | 86.27 | 49.65 | 0.23 | 0.09 | 2.41 | 3.21 | 262.58 |
| **S9_X97** | 104.42 | 143.07 | 1978.73 | 9775.96 | 39.98 | 61.90 | 1.29 | 81.82 | 46.72 | 0.22 | 466.82 | 59.01 | 10.20 | 121.25 |
| **S9_X98** | 28.15 | 52.74 | 759.15 | 6721.45 | 6.89 | 83.39 | 0.51 | 77.55 | 270.72 | 167.31 | 353.28 | 205.54 | 9.17 | 62.97 |
| **S9-10_X99** | 153.03 | 17.06 | 28.23 | 80.77 | 2.87 | 80.45 | 5.17 | 69.61 | 3125.90 | 25.36 | 2913.53 | 2035.85 | 11.59 | 686.99 |
| **S9-10_X100** | 37.04 | 19.56 | 29.21 | 58.56 | 2.92 | 106.55 | 1.77 | 98.28 | 4283.95 | 21.46 | 1306.22 | 2643.76 | 10.64 | 346.77 |
| **S10_X101** | 309.61 | 19.93 | 5.62 | 9.00 | 0.00 | 27.82 | 0.61 | 51.39 | 301.07 | 1.12 | 22.01 | 739.12 | 3.81 | 1915.53 |
| **S10_X102** | 133.26 | 12.80 | 6.22 | 6.05 | 0.04 | 32.14 | 1.19 | 41.12 | 317.32 | 1.60 | 9.65 | 755.04 | 1.99 | 1254.90 |
| **S11_X104** | 1.50 | 6.66 | 2.26 | 0.21 | 0.00 | 5.27 | 0.48 | 18.59 | 0.11 | 0.44 | 0.33 | 0.89 | 0.12 | 118.77 |
| **S11_X105** | 3.31 | 3.21 | 1.77 | 0.40 | 0.00 | 5.63 | 1.72 | 16.03 | 0.04 | 0.73 | 0.03 | 0.00 | 0.11 | 32.29 |
| **S11_X106** | 2.91 | 5.56 | 1.86 | 0.29 | 0.00 | 6.91 | 1.26 | 19.21 | 0.03 | 0.71 | 0.12 | 0.00 | 0.76 | 292.28 |
| **S12 _X107** | 1.26 | 3.30 | 1.91 | 0.61 | 0.00 | 2.65 | 1.43 | 19.72 | 0.01 | 0.08 | 0.06 | 0.00 | 0.00 | 6.54 |
| **S12 _X108** | 2.65 | 6.76 | 2.08 | 0.36 | 0.00 | 9.69 | 0.17 | 16.69 | 0.15 | 0.29 | 0.10 | 0.15 | 0.11 | 1.15 |
| **S12 _X109** | 1.14 | 4.46 | 2.07 | 0.29 | 0.00 | 5.97 | 0.63 | 11.96 | 0.00 | 0.19 | 0.18 | 0.03 | 0.00 | 9.28 |
| **3. Transcriptional levels (RPKM values) of fourteen genes in anther expression data of M6007 genetic background** | | | | | | | | | | | | | | |
| **Stages** | **Zm00001eb025490** | **Zm00001eb223980** | **Zm00001eb035410** | **Zm00001eb317040** | **Zm00001eb147560** | **Zm00001eb185610** | **Zm00001eb028240** | **Zm00001eb278580** | **Zm00001eb073500** | **Zm00001eb294840** | **Zm00001eb250600** | **Zm00001eb387330** | **Zm00001eb026670** | **Zm00001eb033930** |
| **S5_X147** | 208.20 | 143.34 | 280.99 | 0.24 | 0.00 | 20.72 | 1.03 | 35.19 | 129.16 | 0.14 | 0.98 | 71.37 | 0.00 | 161.90 |
| **S5_X148** | 97.76 | 64.73 | 25.46 | 0.67 | 0.00 | 15.03 | 1.86 | 28.96 | 56.56 | 0.00 | 0.26 | 2.09 | 0.26 | 143.05 |
| **S5_X149** | 92.06 | 131.31 | 65.75 | 0.17 | 0.00 | 12.70 | 1.82 | 38.16 | 78.66 | 0.03 | 0.09 | 77.18 | 0.00 | 144.60 |
| **S6_X150** | 189.63 | 109.91 | 752.47 | 0.33 | 0.00 | 13.84 | 3.15 | 32.11 | 202.27 | 0.01 | 0.45 | 0.39 | 0.03 | 203.34 |
| **S6_X151** | 180.78 | 157.41 | 707.00 | 0.20 | 0.05 | 13.67 | 3.73 | 30.42 | 153.83 | 0.08 | 0.48 | 1.19 | 0.03 | 158.75 |
| **S7_X153** | 189.01 | 136.90 | 507.91 | 0.29 | 0.08 | 10.27 | 3.96 | 40.07 | 104.69 | 0.00 | 0.80 | 0.26 | 0.04 | 142.52 |
| **S7_X154** | 169.39 | 165.98 | 638.58 | 0.07 | 0.00 | 17.14 | 4.80 | 42.93 | 166.05 | 0.11 | 0.26 | 0.87 | 0.23 | 289.48 |
| **S8a_X155** | 237.94 | 172.75 | 243.50 | 0.50 | 0.07 | 15.15 | 0.27 | 42.89 | 119.27 | 0.10 | 0.35 | 0.06 | 0.14 | 552.72 |
| **S8a_X156** | 168.89 | 143.47 | 82.26 | 0.82 | 0.07 | 14.41 | 0.23 | 34.10 | 130.41 | 0.09 | 0.03 | 0.00 | 0.33 | 698.03 |
| **S8a_X157** | 293.98 | 145.10 | 147.61 | 1.11 | 0.00 | 18.17 | 0.89 | 55.52 | 95.53 | 0.05 | 0.20 | 1.35 | 0.26 | 728.36 |
| **S8a_X158** | 265.24 | 194.44 | 312.89 | 0.17 | 0.12 | 16.30 | 0.48 | 42.84 | 190.90 | 0.16 | 0.20 | 0.00 | 0.04 | 258.01 |
| **S8b_X159** | 231.09 | 509.54 | 1492.39 | 3562.10 | 6.38 | 22.34 | 0.00 | 83.44 | 159.30 | 1.09 | 0.16 | 0.00 | 0.28 | 937.48 |
| **S8b_X160** | 105.88 | 478.68 | 1078.62 | 1157.80 | 0.00 | 29.54 | 0.03 | 41.27 | 80.29 | 0.01 | 0.06 | 0.00 | 0.18 | 901.80 |
| **S8b_X161** | 123.83 | 302.90 | 517.14 | 626.24 | 0.04 | 20.21 | 0.00 | 45.90 | 124.25 | 0.11 | 0.16 | 0.00 | 0.16 | 994.67 |
| **S8b-9 _X162** | 252.58 | 372.07 | 2078.46 | 8877.70 | 142.76 | 25.65 | 6.28 | 118.37 | 180.73 | 1.21 | 0.41 | 0.70 | 1.09 | 1039.11 |
| **S8b-9 _X164** | 344.97 | 240.84 | 2280.23 | 14395.26 | 16.18 | 43.58 | 15.27 | 189.16 | 159.49 | 0.21 | 0.03 | 0.98 | 7.98 | 1592.42 |
| **S8b-9 _X165** | 381.78 | 320.40 | 1942.56 | 7958.34 | 160.68 | 32.53 | 11.40 | 121.17 | 156.09 | 0.47 | 304.43 | 132.00 | 11.33 | 1004.95 |
| **S9_X166** | 349.45 | 173.15 | 2257.73 | 15280.18 | 1.43 | 25.71 | 32.33 | 158.55 | 136.68 | 0.19 | 0.08 | 1.80 | 8.57 | 1267.49 |
| **S9_X167** | 326.66 | 102.28 | 626.39 | 4835.03 | 103.00 | 39.42 | 11.86 | 101.30 | 756.00 | 0.07 | 5036.98 | 939.77 | 29.40 | 2117.65 |
| **S9-10_X168** | 459.25 | 23.50 | 26.01 | 720.35 | 10.96 | 53.69 | 5.92 | 75.78 | 2519.90 | 0.10 | 4740.34 | 2144.31 | 41.03 | 1971.37 |
| **S9-10_X169** | 424.12 | 36.11 | 196.77 | 851.50 | 8.94 | 44.36 | 5.39 | 104.75 | 1629.00 | 0.06 | 4953.52 | 1636.63 | 58.21 | 1715.57 |
| **S9-10_X170** | 454.09 | 42.56 | 383.75 | 1374.63 | 11.60 | 29.52 | 4.70 | 120.71 | 909.84 | 0.08 | 4774.89 | 1296.42 | 54.06 | 1619.98 |
| **S10_X171** | 246.66 | 20.77 | 3.24 | 3.50 | 0.00 | 10.10 | 0.09 | 52.18 | 61.32 | 0.32 | 4.87 | 586.89 | 3.18 | 4961.71 |
| **S10_X172** | 445.69 | 20.79 | 4.61 | 8.38 | 0.00 | 10.03 | 0.06 | 65.12 | 81.15 | 0.55 | 14.96 | 653.32 | 3.50 | 3156.68 |
| **S10_X173** | 55.84 | 18.63 | 4.68 | 5.25 | 0.00 | 7.94 | 0.29 | 79.50 | 38.89 | 1.07 | 1.24 | 663.62 | 3.78 | 3041.13 |
| **S11_X174** | 4.90 | 8.58 | 1.38 | 0.02 | 0.00 | 4.01 | 0.09 | 21.91 | 0.85 | 0.19 | 0.34 | 0.03 | 0.04 | 984.99 |
| **S11_X175** | 7.98 | 3.85 | 3.04 | 0.00 | 0.00 | 1.90 | 0.55 | 7.29 | 0.44 | 0.25 | 0.00 | 0.00 | 0.00 | 1929.37 |
| **S11_X176** | 6.95 | 9.40 | 2.17 | 0.02 | 0.00 | 6.40 | 0.56 | 48.57 | 1.23 | 0.20 | 1.62 | 0.06 | 0.00 | 2548.43 |
| **S12_X177** | 2.09 | 7.68 | 1.64 | 0.20 | 0.00 | 2.69 | 0.20 | 18.80 | 0.11 | 0.14 | 0.22 | 0.00 | 0.00 | 358.16 |
| **S12_X178** | 2.01 | 6.90 | 1.17 | 0.18 | 0.00 | 3.05 | 1.24 | 20.68 | 0.25 | 0.09 | 0.34 | 0.00 | 0.00 | 229.82 |
| **S12_X179** | 0.41 | 4.85 | 2.12 | 2.20 | 0.04 | 1.38 | 0.92 | 20.66 | 0.72 | 0.14 | 0.17 | 0.00 | 0.00 | 204.51 |

**Table S2:** The detailed cutin, wax and internal lipid compositions in WT, *pksb* and *tkpr1-1/-2* anthers.

| **Cutin constituents** | **Amount: Mean ± SD (μg/mm^2^)** | | | **Up (folds)** | | **Down (folds)** | |
| --- | --- | --- | --- | --- | --- | --- | --- |
|  | **Wild Type** | ***pksb*** | ***tkpr1-1/-2*** | ***pksb*** | ***tkpr1-1/-2*** | ***pksb*** | ***tkpr1-1/-2*** |
| **C14 FA** | 0.000177±0.000017 | 0.000326±0.000072 | 0.000283±0.000065 | 1.841808 | 1.598870 | N | N |
| **C16 FA** | 0.259763±0.015337 | 0.031572±0.005003 | 0.013986±0.0032695 | N | N | 8.227638 | 18.57307 |
| **C18 FA** | 0.004488±0.000847 | 0.004082±0.000467 | 0.004613±0.000745 | N | 1.027852 | 1.099461 | N |
| **C18:1 FA** | 0.000869±0.000151 | 0.000075±0.000013 | 0.000268±0.000079 | N | N | 11.648794 | 3.242537 |
| **C18:2 FA** | 0.045721±0.003090 | 0.016770±0.001405 | 0.012545±0.003616 | N | N | 2.726357 | 3.644560 |
| **C18:3 FA** | 0.0718910±0.0111814 | 0.006122±0.001219 | 0.000989±0.000303 | N | N | 11.743058 | 72.69059 |
| **C20 FA** | 0.003971±0.000905 | 0.002733±0.000203 | 0.005604±0.000190 | N | 1.411231 | 1.452982 | N |
| **C22 FA** | 0.001901±0.000273 | 0.001605±0.000101 | 0.002871±0.000234 | N | 1.510258 | 1.184424 | N |
| **C24 FA** | 0.000670±0.000098 | 0.001708±0.000192 | 0.002429±0.0002142 | 2.549254 | 3.625373 | N | N |
| **C26 FA** | 0.000113±0.000016 | 0.000492±0.000140 | 0.000935±0.000079 | 4.353982 | 8.274336 | N | N |
| **C16 ALC** | 0.000280±0.000011 | 0.000293±0.000017 | 0.000478±0.000044 | 1.050179 | 1.713262 | N | N |
| **C22 ALC** | 0.000089±0.000017 | 0.000304±0.000046 | 0.000400±0.000082 | 3.416009 | 4.509583 | N | N |
| **C22 HFA** | 0.002191±0.000229 | 0.003474±0.000031 | 0.005261±0.000450 | 1.585577 | 2.401187 | N | N |
| **C24 HFA** | 0.005792±0.000453 | 0.009212±0.000716 | 0.007167±0.000586 | 1.590470 | 1.237396 | N | N |
| **C26 HFA** | 0.0014872±0.000204 | 0.002595±0.000220 | 0.002667±0.000371 | 1.745124 | 1.793544 | N | N |
| **C18 HFA** | 0.001178±0.0001676 | 0.001289±0.000299 | 0.001676±0.000097 | 1.094228 | 1.422750 | N | N |
| **C16 ωHFA** | 0.138266±0.033239 | 0.218799±0.044112 | 0.064339±0.016407 | 1.582450 | N | N | 2.149023 |
| **C18:1 ωHFA** | 0.005566±0.001384 | 0.010616±0.003318 | 0.003869±0.000194 | 1.907294 | N | N | 1.438615 |
| **C18:2 ωHFA** | 0.001031±0.000246 | 0.001475±0.000285 | 0.001419±0.000255 | 1.430650 | 1.376334 | N | N |
| **C22 DFA** | 0 | 0 | 0.000041±0.000013 | N | 1.000000 | N | N |
| **C18-9,10 DHDFA** | 0.247490±0.100091 | 0.660976±0.085278 | 0.139703±0.012930 | 2.670718 | N | N | 1.771544 |
|  | | | | | | | |
| **Wax constituents** | **Amount: Mean ± SD (μg/mm^2^)** | | | **Up (folds)** | | **Down (folds)** | |
|  | **Wild Type** | ***pksb*** | ***tkpr1-1/-2*** | ***pksb*** | ***tkpr1-1/-2*** | ***pksb*** | ***tkpr1-1/-2*** |
| **C21 ALK** | 0 | 0 | 0.000081±0.000033 | 0 | 1.000000 | 0 | N |
| **C24 ALK** | 0 | 0 | 0.000102±0.000031 | 0 | 1.000000 | 0 | N |
| **C25 ALK** | 0.001209±0.000353 | 0.013394±0.001325 | 0.021170±0.004098 | 11.08324 | 17.51770 | N | N |
| **C26 ALK** | 0.000127±0.000023 | 0.000946±0.000273 | 0.001194±0.000091 | 7.476075 | 9.434584 | N | N |
| **C27 ALK** | 0.007458±0.000354 | 0.032325±0.005776 | 0.031064±0.000500 | 4.334140 | 4.165056 | N | N |
| **C28 ALK** | 0.000557±0.000084 | 0.001373±0.000249 | 0.001091±0.000109 | 2.462292 | 1.956872 | N | N |
| **C29 ALK** | 0.0085372±0.000555 | 0.024138±0.003732 | 0.013683±0.002210 | 2.827413 | 1.602807 | N | N |
| **C30 ALK** | 0.000538±0.000058 | 0.001276±0.000465 | 0.000742±0.000073 | 2.369615 | 1.378463 | N | N |
| **C31 ALK** | 0.024394±0.003826 | 0.037468±0.012368 | 0.005391±0.000505 | 1.535992 | N | N | 4.524761 |
| **C32 ALK** | 0.000130±0.000021 | 0.000279±0.000093 | 0.000188±0.000041 | 2.145249 | 1.442810 | N | N |
| **C33 ALK** | 0.001221±0.000226 | 0.002376±0.000547 | 0.000628±0.000142 | 1.946014 | N | N | 1.944902 |
| **C34 ALK** | 0.000116±0.000023 | 0.000228±0.000069 | 0.000093±0.000028 | 1.967807 | N | N | 1.248581 |
| **C16 FA** | 0.001330±0.000150 | 0.001054±0.000113 | 0.000334±0.000040 | N | N | 1.262064 | 3.979059 |
| **C18 FA** | 0.000809±0.000117 | 0.000763±0.000136 | 0.000400±0.000081 | N | N | 1.060664 | 2.024647 |
| **C18:1 FA** | 0.000585±0.000014 | 0.000982±0.000487 | 0.000142±0.000032 | 1.677457 | N | N | 4.109192 |
| **C18:2 FA** | 0.000246±0.000065 | 0.000385±0.000135 | 0.000161±0.000044 | 1.567765 | N | N | 1.526139 |
| **C20 FA** | 0.000079±0.000035 | 0.000156±0.000090 | 0.000342±0.000312 | 1.987985 | 4.354767 | N | N |
| **C24 FA** | 0 | 0.000104±0.000056 | 0.000134±0.000078 | 1.000000 | 1.300000 | N | N |
| **C26 FA** | 0 | 0 | 0.000102±0.000048 | N | 1.000000 | N | N |
| **C28 FA** | 0.000290±0.000037 | 0.000259±0.000090 | 0.000202±0.000147 | N | N | 1.118901 | 1.435530 |
|  | | | | | | | |
| **TFA constituents** | **Amount: Mean ± SD (μg/mm^2^)** | | | **Up (folds)** | | **Down (folds)** | |
|  | **Wild Type** | ***pksb*** | ***tkpr1-1/-2*** | ***pksb*** | ***tkpr1-1/-2*** | ***pksb*** | ***tkpr1-1/-2*** |
| **C14 FA** | 0.029704±0.001050 | 0.086310±0.005661 | 0.061646±0.002110 | 2.905611 | 2.075316 | N | N |
| **C16 FA** | 8.331868±0.823203 | 5.142659±0.616012 | 1.808165±0.2539631 | N | N | 1.620148 | 4.607913 |
| **C16:1 FA** | 0.014976±0.000236 | 0.016183±0.004470 | 0.030510±0.014389 | 1.080542 | 2.037231 | N | N |
| **C18 FA** | 0.354282±0.024181 | 0.950605±0.056599 | 1.071219±0.084980 | 2.683188 | 3.023632 | N | N |
| **C18:1 FA** | 0.096818±0.014321 | 0.101363±0.027886 | 0.040345±0.023495 | 1.046939 | N | N | 2.399768 |
| **C18:2 FA** | 3.737308±0.560431 | 3.136609±0.512980 | 1.629762±0.382414 | N | N | 1.191512 | 2.293161 |
| **C18:3 FA** | 3.249283±0.169159 | 1.308376±0.178204 | 0.208590±0.027767 | N | N | 2.483448 | 15.57740 |
| **C20 FA** | 0.371248±0.031705 | 1.044261±0.082254 | 1.644525±0.243314 | 2.812840 | 4.429723 | N | N |
| **C20:1 FA** | 0.015980±0.001564 | 0.018555±0.004498 | 0.022980±0.006361 | 1.161128 | 1.438026 | N | N |
| **C22 FA** | 0.110715±0.002455 | 0.361302±0.025780 | 0.693379±0.069566 | 3.263346 | 6.262728 | N | N |
| **C24 FA** | 0.065432±0.004437 | 0.326216±0.016944 | 0.660452±0.059268 | 4.985590 | 10.09376 | N | N |
| **C26 FA** | 0.077974±0.005582 | 0.224450±0.056369 | 0.378492±0.057878 | 2.878539 | 4.854112 | N | N |
| **C28 FA** | 0.160028±0.026045 | 0.370250±0.038609 | 0.7216674±0.275056 | 2.313659 | 4.509637 | N | N |

**Table S3:** Primers used in this study.

| **Construct or gene** | **Primer name** | **Sequence (5’ to 3’)** |
| --- | --- | --- |
| **Plant transformation** | | |
| pCas9-ZmTKPR1-2 | ZmTKPR1-2-MT1-F | ATATATGGTCTCTGGCGAAGCCGTTTGATAAGCCAAGGTTTTAGAGCTAGAAATAGCAA |
|  | ZmTKPR1-2-MT2-R | ATTATTGGTCTCTAAACGGCAAGGTATGTGTAACCGTGCTTCTTGGTGCCGC |
| pCas9-ZmTKPR1-1 | ZmTKPR1-1-MT1-F | ATATATGGTCTCTGGCGAAGCCGCTTGACGAGCCAGGGTTTTAGAGCTAGAAATAGCAA |
|  | ZmTKPR1-1-MT2-R | ATTATTGGTCTCTAAACGACCATTCCATCTCTCTCATGCTTCTTGGTGCCGC |
| pCas9-Zm00001eb223980 | Zm00001eb223980-MT1-F | ATATATGGTCTCTGGCGAGTAATATTGACAATGAAGTGTTTTAGAGCTAGAAATAGCAA |
|  | Zm00001eb223980-MT2-R | ATTATTGGTCTCTAAACTCAGAGATTAAATGACCAGTGCTTCTTGGTGCCGC |
| pCas9-Zm00001eb073500 | Zm00001eb073500-MT1-F | ATATATGGTCTCTGGCGACCGACACGAAGCGCTGCGCGTTTTAGAGCTAGAAATAGCAA |
|  | Zm00001eb073500-MT2-R | ATTATTGGTCTCTAAACGCCAGCGACGACTACGTGCTGCTTCTTGGTGCCGC |
| pCas9-Zm00001eb025490 | Zm00001eb025490-MT1-F | ATATATGGTCTCTGGCGACAGCTCGTCCACCGCCTCGGTTTTAGAGCTAGAAATAGCAA |
|  | Zm00001eb025490-MT2-R | ATTATTGGTCTCTAAACAGCACGTCCGCCTTCTCCGTGCTTCTTGGTGCCGC |
| pCas9-Zm00001eb250600 | Zm00001eb250600-MT1-F | ATATATGGTCTCTGGCGACCACGGGCCCAGCGCCGGGGTTTTAGAGCTAGAAATAGCAA |
|  | Zm00001eb250600-MT2-R | ATTATTGGTCTCTAAACCTGCTGAGCCAGCAGCACCTGCTTCTTGGTGCCGC |
| pCas9-Zm00001eb028240 | Zm00001eb028240-MT3-F | ATATATGGTCTCTGGCGATTACCTTCAAAATGACATGGTTTTAGAGCTAGAAATAGCAA |
|  | Zm00001eb028240-MT4-R | ATTATTGGTCTCTAAACGTGTATATGTGTGTGCAGC TGCTTCTTGGTGCCGC |
| pCas9-Zm00001eb026670 | Zm00001eb026670MT1-F | ATATATGGTCTCTGGCGAGTGAGCAGCAGCACCGCCG GTTTTAGAGCTAGAAATAGCAA |
|  | Zm00001eb026670-MT2-R | ATTATTGGTCTCTAAACCGAGTATGCCGCCGCGACGTGCTTCTTGGTGCCGC |
| pCas9-Zm00001eb294840 | Zm00001eb294840-MT1-F | ATATATGGTCTCTGGCGACATGTCCTCGCCGACGAAGGTTTTAGAGCTAGAAATAGCAA |
|  | Zm00001eb294840-MT2-R | ATTATTGGTCTCTAAACCGTACCTGAAGACCCACGGTGCTTCTTGGTGCCGC |
| pCas9-Zm00001eb278580 | Zm00001eb278580-MT1-F | ATATATGGTCTCTGGCGAGAGGCAATCATCAAGGCGGGTTTTAGAGCTAGAAATAGCAA |
|  | Zm00001eb278580-MT2-R | ATTATTGGTCTCTAAACACTACGACCGCCTGCTAGTTGCTTCTTGGTGCCGC |
| pCas9-Zm00001eb387330 | Zm00001eb387330-MT1-F | ATATATGGTCTCTGGCGACGAAGCTGCTGCCATGGCGGTTTTAGAGCTAGAAATAGCAA |
|  | Zm00001eb387330-MT2-R | ATTATTGGTCTCTAAACTATGACGACGACGCTGTGGTGCTTCTTGGTGCCGC |
| pCas9-Zm00001eb147560 | Zm00001eb147560-MT1-F | ATATATGGTCTCTGGCGAGCATTGATCTCGGCGTACA GTTTTAGAGCTAGAAATAGCAA |
|  | Zm00001eb147560-MT2-R | ATTATTGGTCTCTAAACGCTGACGCGACGGCTGTCCTGCTTCTTGGTGCCGC |
| pCas9-Zm00001eb033930 | Zm00001eb033930-MT1-F | ATATATGGTCTCTGGCGAACGGCGGTGTTCTTTCACGGTTTTAGAGCTAGAAATAGCAA |
|  | Zm00001eb033930-MT2-R | ATTATTGGTCTCTAAACCGCGGCAGGAATCCCAGCGTGCTTCTTGGTGCCGC |
| pCas9-Zm00001eb185610 | Zm00001eb185610-MT1-F | ATATATGGTCTCTGGCGAAGACACGCTTCTCTGTCAAGTTTTAGAGCTAGAAATAGCAA |
|  | Zm00001eb185610-MT2-R | ATTATTGGTCTCTAAACGTTGGCCTGACCGTGCCAATGCTTCTTGGTGCCGC |
| **Transformation identification** | | |
| Bar | Bar-F | TCTACCATGAGCCCAGAAC |
|  | Bar-R | TCAAATCTCGGTGACGGGCA |
| **Genotype identification for T_0_, F_1_, and F_2_ plants** | | |
| ZmTKPR1-1 | ZmTKPR1-1-T-F | AGAATGGCTAACACTGCTAAG |
|  | ZmTKPR1-1-T-R | GAAGCTGCAAATATGAGAAAC |
| ZmTKPR1-2 | ZmTKPR1-2-T-F | AGAATGTTCCTGCCGATGCTT |
|  | ZmTKPR1-2-T-R | GCAAATACCTGGGTCCCTGAC |
| Zm00001eb223980 | Zm00001eb223980-T-F | CGTGATCGTCAGTGGCATAT |
|  | Zm00001eb223980-T-R | GTTGTTCTCTCAGTAAGCACAGA |
| Zm00001eb073500 | Zm00001eb073500-T-F | TAGTACGTGGCGTGAACTACAC |
|  | Zm00001eb073500-T-R | CCTTGGTCCCGTCGAAGTG |
| Zm00001eb025490 | Zm00001eb025490-T-F | ATGACGGCCCCGGAGAAGGC |
|  | Zm00001eb025490-T-R | ACCATGTTGCCCACCAGG |
| Zm00001eb250600 | Zm00001eb250600-T-F2 | TGGCCCACTTCTCGGACG |
|  | Zm00001eb250600-T-R2 | ACCTGCAACCGTTCCTTAAT |
| Zm00001eb028240 | Zm00001eb028240-T-F | ATTGGTCTCCTTGATTTAACAGGA |
|  | Zm00001eb028240-T-R | CTCGATGCTGAAATTGCCGC |
| Zm00001eb026670 | Zm00001eb026670-T-F | CGTCAACCTCAAGTACGTCC |
|  | Zm00001eb026670-T-R | CCCGTGCTCTCTGTCATGTC |
| Zm00001eb294840 | Zm00001eb294840-T-F | GAGTAGAAGTGCCAGCCAGC |
|  | Zm00001eb294840-T-R | CATCTTTCAGTAACCCACCGC |
| Zm00001eb278580 | Zm00001eb278580-T-F | GGAACGATGTAGACGCTTACT |
|  | Zm00001eb278580-T-R | AAGCCATCAAAGGTCACGAA |
| Zm00001eb387330 | Zm00001eb387330-T-F | ACAGACCTATCGCTACACAGTC |
|  | Zm00001eb387330-T-R | CACGTACAGCAGTAGTCTAACGA |
| Zm00001eb147560 | Zm00001eb147560-T-F | TCCTGCACTTGCACCCCTAT |
|  | Zm00001eb147560-T-R | GACGATCCAGTCGTGAACCC |
| Zm00001eb033930 | Zm00001eb033930-T-F | CCCTGTCGGTTCTCCTCTCC |
|  | Zm00001eb033930-T-R | GTCGTGAACGGGATGGAGTC |
| Zm00001eb185610 | Zm00001eb185610-T-F | TGTGCCAGAAGCTCGGTCT |
|  | Zm00001eb185610-T-R | CCTGAAGACACACCGCAGC |
| **qRT-PCR** | | |
| ZmTKPR1-1 | qTKPR1-1-F | GAGAGACGGGGAAGTTCACG |
|  | qTKPR1-1-R | GCTGCAGATGTACCTCCCTC |
| ZmTKPR1-2 | qTKPR1-2-F | CAGGGACCCAGGAAATCACC |
|  | qTKPR1-2-R | ACAGATCAGCTCGCACGATT |
| Zm00001eb223980 | qZm00001eb223980-F | GCTTACAGGGATCAGTTGATTCG |
|  | qZm00001eb223980-R | TTCTTGCTATGCTGGTGCGA |
| Zm00001eb073500 | qZm00001eb073500-F | CAACGCCTCGCCAGGAAC |
|  | qZm00001eb073500-R | CATCACGGTTTTGTTCCCTCT |
| Zm00001eb025490 | qZm00001eb025490-F | CTACTACATGTTCGACGGCC |
|  | qZm00001eb025490-R | TGTCGACGAAGAACTGGACG |
| Zm00001eb250600 | qZm00001eb250600-F | CGGCTTCTCTTCTGAGCTCA |
|  | qZm00001eb250600-R | GCGTCCTTCATGAACGTGTAG |
| ZmZm00001eb028240 | qZm00001eb028240-F | GAGTTAACACCATATCAGCAGATGA |
|  | qZm00001eb028240-R | CCCATTGTATTGAGTCCATTGTCA |
| Zm00001eb026670 | qZm00001eb026670-F | ATCCGGATTCAAGTGCAACAG |
|  | qZm00001eb026670-R | TAGCTCTGAATGCTGTCCACC |
| Zm00001eb294840 | qZm00001eb294840-F | ATCACAAGAGGAGTCCTGTGC |
|  | qZm00001eb294840-R | AGGTACCACGACAGGAAGCC |
| Zm00001eb278580 | qZm00001eb278580-F | CATGGGTGAAGGAGCTGGAG |
|  | qZm00001eb278580-R | TCACAGTTCACAGCACCTCC |
| Zm00001eb387330 | qZm00001eb387330-F | CGTCGAGTCCATGATGAGGG |
|  | qZm00001eb387330-R | TGACATCCACCACCTCTTGC |
| Zm00001eb147560 | qZm00001eb147560-F | GTTCATGAAGAACGGCCTCC |
|  | qZm00001eb147560-R | TGCAGTTGTGGTCGAGATCA |
| Zm00001eb033930 | qZm00001eb033930-F | GCCAAGAACGTGAAACCCTC |
|  | qZm00001eb033930-R | TAGCATAGCACAGCACAGCA |
| Zm00001eb185610 | qZm00001eb185610-F | GGCGCAACTTGATCTGTTCC |
|  | qZm00001eb185610-R | AGAACGTGGTCACCGACTTG |
| ZmPKSB | qPKSB-F | TCTGCTTCCGTGTGATTGCT |
|  | qPKSB-R | GTGAATTTCGGGGCCCCTTA |
| ZmMYB84 | qMYB84-F | CGGAGAAAGGCAACGACAGC |
|  | qMYB84-R | CGACTTCGCACGAACGGTATT |
| ZmKCAS-4 | qKCAS-4-F | GCGTCTGGATGATCGGATTC |
|  | qKCAS-4-R | CTTGAGCACGTCGGGAATG |
| ZmGL8 | qGL8-F | CCATTCTTCCCGAGTCCCTTATC |
|  | qGL8-R | GAGGAAATGTGCGAATCTGAACA |
| ZmCER8 | qCER8-F | GCTCACATCCTTGACCGCAT |
|  | qCER8-R | TCCACAATGTCGTCCCTCAA |
| ZmABCG26 | qABCG26-F | GCGGAGGACGATCATCACG |
|  | qABCG26-R | CCCAGCGAGGAGAAGTGGT |
| ZmABCG2a | qABCG2a-F | GGCGAGTTCATCCTGGAGAA |
|  | qABCG2a-R | TAGACGACGACCATGCAGAGC |
| ZmHCT12 | qHCT12-F | CACCCATCCAGTTCGTTGA |
|  | qHCT12-R | TAATCCAACACGCACGCCTA |
| ZmLTL1 | qLTL1-F | GCTGGTGGACATGATCAAGG |
|  | qLTL1-R | AAGTCCATGTGCATCCGGTA |
| ZmCER1 | qCER1-F | ACGAGGAACAGAAGATGGCA |
|  | qCER1-R | AGTTCTCGCAGGCATGGATA |
| ZmGL2 | qGL2-F | TACTCCTTCCACGTCAGCGA |
|  | qGL2-R | ACGTCGCCCCTGATCTTG |
| ZmCL3929 | qCL3929-F | TCACCTTACCCACCTTACCAGC |
|  | qCL3929-R | CCACATCATCCGCACATACC |
| ZmUbi2 | Ubi2-F | CGACAACGTGAAGGCGAAGA |
|  | Ubi2-R | ACGCAGATACCCAGGTACAGC |
| ZmCyanase | Cyanase-F | GCTGGTGAGGAGGAGAAACA |
|  | Cyanase-R | CAGCAATCATGCCAGGTAGA |
| **Transient dual-luciferase assay** | | |
| proZmTKPR1-1:LUC | pZmTKPR1-1-LUC-F | ATTACGCCAAGCTTGGTACCGAGAGTATCAGCGACTCCCT |
|  | pZmTKPR1-1-LUC-R | TTGGCGTCTTCCATGGATCCCATTCTCTCAGACTCGAGGTG |
| proZmTKPR1-2:LUC | pZmTKPR1-2-LUC-F | ATTACGCCAAGCTTGGTACCTCATACGCCATGGTGAGCTCTTCG |
|  | pZmTKPR1-2-LUC-R | TTGGCGTCTTCCATGGATCCGCCCCGGTTACACATACCTTGC |
| 35S-Ω:ZmMYB84 | p35S-Ω-MYB84-F | ATTACGCCAAGCTTGGTACCGGCCTCCGTGTTCTTTCTGA |
|  | p35S-Ω-MYB84-R | TTGGCGTCTTCCATGGATCCCCTCGCCGGGCTGCTCTCGC |
| **BiFC assay and Co-IP assay** | | |
| 35S:PKSB-nYFP-FLAG | PKSB-nYFP-FLAG-F | CTCGGTACCCTCGAGATGGTGAGCAGCAGCATGGA |
|  | PKSB-nYFP-FLAG-R | GCTCACCATTTCGAAGAGCTGGGCTGAGGCGCGCG |
| 35S:PKSB-cYFP-MYC | PKSB-cYFP-MYC-F | CTCGGTACCCTCGAGATGGTGAGCAGCAGCATGGA |
|  | PKSB-cYFP-MYC-R | CTGCTTGTCTTCGAAGAGCTGGGCTGAGGCGCGCG |
| 35S:TKPR1-1-nYFP-FLAG | TKPR1-1-nYFP-FLAG-F | CTCGGTACCCTCGAGATGGCTAACACTGCTAAGGG |
|  | TKPR1-1-nYFP-FLAG-R | GCTCACCATTTCGAACGCGTGCCGGCGTTCCTCGG |
| 35S:TKPR1-1-cYFP-MYC | TKPR1-1-cYFP-MYC-F | CTCGGTACCCTCGAGATGGCTAACACTGCTAAGGG |
|  | TKPR1-1-cYFP-MYC-R | CTGCTTGTCTTCGAACGCGTGCCGGCGTTCCTCGG |
| 35S:TKPR1-2-nYFP-FLAG | TKPR1-2-nYFP-FLAG-F | CTCGGTACCCTCGAGATGGTGACCTCAAGCAAGGG |
|  | TKPR1-2-nYFP-FLAG-R | GCTCACCATTTCGAACAGGGGGCACTCCAGCAGGT |
| 35S:TKPR1-2-cYFP-MYC | TKPR1-2-cYFP-MYC-F | CTCGGTACCCTCGAGATGGTGACCTCAAGCAAGGG |
|  | TKPR1-2-cYFP-MYC-R | CTGCTTGTCTTCGAACAGGGGGCACTCCAGCAGGT |
| **Yeast two-hybrid assay** | | |
| pGADT7-PKSB | PKSB-AD-F | CTCGGTACCCTCGAGATGGTGAGCAGCAGCATGGA |
|  | PKSB-AD-R | GCTCACCATTTCGAAGAGCTGGGCTGAGGCGCGCG |
| pGBKT7-PKSB | PKSB-BK-F | CTCGGTACCCTCGAGATGGTGAGCAGCAGCATGGA |
|  | PKSB-BK-R | CTGCTTGTCTTCGAAGAGCTGGGCTGAGGCGCGCG |
| pGADT7-TKPR1-1 | TKPR1-1-AD-F | CTCGGTACCCTCGAGATGGCTAACACTGCTAAGGG |
|  | TKPR1-1-AD-R | GCTCACCATTTCGAACGCGTGCCGGCGTTCCTCGG |
| pGBKT7-TKPR1-1 | TKPR1-1-BK-F | CTCGGTACCCTCGAGATGGCTAACACTGCTAAGGG |
|  | TKPR1-1-BK-R | CTGCTTGTCTTCGAACGCGTGCCGGCGTTCCTCGG |
| pGADT7-TKPR1-2 | TKPR1-2-AD-F | CTCGGTACCCTCGAGATGGTGACCTCAAGCAAGGG |
|  | TKPR1-2-AD-R | GCTCACCATTTCGAACAGGGGGCACTCCAGCAGGT |
| pGBKT7-TKPR1-2 | TKPR1-2-BK-F | CTCGGTACCCTCGAGATGGTGACCTCAAGCAAGGG |
|  | TKPR1-2-BK-R | CTGCTTGTCTTCGAACAGGGGGCACTCCAGCAGGT |
| pGBKT7-TKPR1-1-9M | 1-F | TCGTCACCGGAGCCAATTCCAA |
|  | 1-R | TTGGAATTGGCTCCGGTGACGA |
|  | 2-F | CGAAAGTCTCGCCATATGGTACG |
|  | 2-R | CGTACCATATGGCGAGACTTTCG |
|  | 3-F | GAGAGACGGCCAAGTTCGCCACGTACGGG |
|  | 3-R | CCCGTACGTGGCGAACTTGGCCGTCTCTC |
|  | 4-F | TCCCGAGGGCCCTGCCCGCCGCCTACGGCGCCCAGTCGTA |
|  | 4-R | TACGACTGGGCGCCGTAGGCGGCGGGCAGGGCCCTCGGGA |
|  | 5-F | ATGGCTAACACTGCTAAGGG |
|  | 5-R | CGCGTGCCGGCGTTCCTCGG |
| pGBKT7-TKPR1-2-7M | 1-F | ATGGTGACCTCAAGCAAGGG |
|  | 1-R | TGCTAGTAGAGGCTGGGGCAGCGAGGACGG |
|  | 2-F | CCGTCCTCGCTGCCCCAGCCTCTACTAGCA |
|  | 2-R | GAACCTTGCGGCGTCGCCGGCGAATAGGCCT |
|  | 3-F | AGGCCTATTCGCCGGCGACGCCGCAAGGTTC |
|  | 3-R | TGTTCAGCCTGGCGGGTATGGGGA |
|  | 4-F | TTCCCCATACCCGCCAGGCTGAACA |
|  | 4-R | CAGGGCGCACTCCAGCAGGT |
| pGADT7-TKPR1-1-4M | 1-F | ATGGCTAACACTGCTAAGGG |
|  | 1-R | TCTCCTCAGCGGAATTAGAT |
|  | 2-F | ATCTAATTCCGCTGAGGAGA |
|  | 2-R | CGGCGTAAGCTATCTGGAGA |
|  | 3-F | TCTCCAGATAGCTTACGCCG |
|  | 3-R | AGGAGCCTGCCCCGCGCCGCCGGCGAGCAGTCGTA |
|  | 4-F | TACGACTGCTCGCCGGCGGCGCGGGGCAGGCTCCT |
|  | 4-R | CGCGTGCCGGCGTTCCTCGG |
| **Construction of the prokaryotic expression vectors** | | |
| ZmTKPR1-1-MBP | ZmTKPR1-1-MBP-F | TATCCACTTCCAATGCGCTACTACGCGTGCCGGCGTTCCT |
|  | ZmTKPR1-1-MBP-R | TGTACTTCCAATCCAATGCGATGGCTAACACTGCTAAGGG |
| ZmTKPR1-1-12 | ZmTKPR1-1-12-F | CTCGTCAAGCGGCTTCTTGAGTCTG |
|  | ZmTKPR1-1-12-R | AAGCCGCTTGACGAGGAAACCAGACGCCCCAGTG |
| ZmTKPR1-1-SA | ZmTKPR1-1-SA-F | GACGACGCGGATGTCAGGACAACCC |
|  | ZmTKPR1-1-SA-R | GACATCCGCGTCGTCGACAGTGAGG |
| ZmTKPR1-1-YA | ZmTKPR1-1-YA-F | ACGGCGGCCCATATCTGGAGACTTT |
|  | ZmTKPR1-1-YA-R | GATATGGGCCGCCGTCGCAAAGATC |
| ZmTKPR1-1-KA | ZmTKPR1-1-KA-F | AGGATCGCTGCGACGGCGTACCATA |
|  | ZmTKPR1-1-KA-R | CGTCGCAGCGATCCTCGCCGAGAAG |
| ZmTKPR1-2-MBP | ZmTKPR1-2-MBP-F | TATCCACTTCCAATGCGCTATCACAGGGGGCACTCCAGCA |
|  | ZmTKPR1-2-MBP-R | TGTACTTCCAATCCAATGCGATGGTGACCTCAAGCAAGGG |
| ZmTKPR1-2-3 | ZmTKPR1-2-3-F | AGGAGCCGTTTGATAAAAGAGGCAACAAAGCCTGA |
|  | ZmTKPR1-2-3-R | TTATCAAACGGCTCCTCGAGTCTGG |
| ZmTKPR1-2-SA | ZmTKPR1-2-SA-F | GACGACGCAGACGTAAGGACGACCC |
|  | ZmTKPR1-2-SA-R | TACGTCTGCGTCGTCTGCGGTGAGG |
| ZmTKPR1-2-YA | ZmTKPR1-2-YA-F | TAGGGCAGCCCATAGATGCATCTTC |
|  | ZmTKPR1-2-YA-R | CTATGGGCTGCCCTAGCCAAGGTAT |
| ZmTKPR1-2-KA | ZmTKPR1-2-KA-F | AATACCGCGGCTAGGGCATACCATA |
|  | ZmTKPR1-2-KA-R | CCTAGCCGCGGTATTTGCAGAGAAA |
| **Electrophoretic mobility shift assay** | | |
| MYB84-Pr | MYB84-Pr-F | TACTTCCAATCCAATGCGATGGGGCGGATCCCGTGCTG |
|  | MYB84-Pr-R | TTATCCACTTCCAATGCGCTATCACACCATGTGATTGGTCA |
| ZmTKPR1-1bio | ZmTKPR1-1bio-F | TAGGAAGGAAGAAGGTTCCTGCAATGCATGTAAGGTTGGTAATTCATGCCTT |
|  | ZmTKPR1-1bio-R | AAAGGCATGAATTACCAACCTTACATGCATTGCAGGAACCTTCTTCCTTCCT |
| ZmTKPR1-2bio | ZmTKPR1-2bio-F | ATTGCAAATCTACCCCCAACCAACCCAGCTTTGTATCTG |
|  | ZmTKPR1-2bio-R | GCAGATACAAAGCTGGGTTGGTTGGGGGTAGATTTGCAA |
| ZmTKPR1-1bio-mt | ZmTKPR1-1bio-mt-F | TAGGAAGGAAGAAGGTTCCTGCAATGCATGTAAGTTTTGTAATTCATGCCTT |
|  | ZmTKPR1-1bio-mt-R | AAGGCATGAATTACAAAACTTACATGCATTGCAGGAACCTTCTTCCTTCCTA |
| ZmTKPR1-2bio-mt | ZmTKPR1-2bio-mt-F | ATTGCAAATCTACCCCCAACAAAACCAGCTTTGTATCTG |
|  | ZmTKPR1-2bio-mt-R | CAGATACAAAGCTGGTTTTGTTGGGGGTAGATTTGCAAT |
| **Subcellular localization in maize protoplasts and tobacco leaves** | | |
| ZmTKPR1-1-GFP | ZmTKPR1-1-GFP-F | ACGAGCTCGGTACCCTCGAGATGGCTAACACTGCTAAGGG |
|  | ZmTKPR1-1-GFP-R | AGCTCCTCGCCCTTGCTCACTCTAGACGCGTGCCGGCGTTCCTCGG |
| ZmTKPR1-2-GFP | ZmTKPR1-2-GFP-F | ACGAGCTCGGTACCCTCGAGATGGTGACCTCAAGCAAGGG |
|  | ZmTKPR1-2-GFP-R | AGCTCCTCGCCCTTGCTCACTCTAGACAGGGGGCACTCC |
| ZmTKPR1-1-mCherry | ZmTKPR1-1-mCherry -F | TCGACGACAAGACCGTCACCATGGCTAACACTGCTAAGGG |
|  | ZmTKPR1-1-mCherry -R | TCGAGTGAGGAGAAGAGCCGCGCGTGCCGGCGTTCCTCGG |
| ZmTKPR1-2-mCherry | ZmTKPR1-2-mCherry -F | TCGACGACAAGACCGTCACCATGGTGACCTCAAGCAAGGG |
|  | ZmTKPR1-2-mCherry -R | TCGAGTGAGGAGAAGAGCCGCAGGGGGCACTCCAGCAGGT |
